# Passive Morphing in Insect Wings: From Biological Mechanisms to Aerospace Structures

**DOI:** 10.3390/biomimetics11100700

**Published:** 2026-10-02

**Authors:** Raluca Andreea Roșu, Oana Dumitrescu, Emilia Georgiana Prisăcariu

**Affiliations:** Romanian Research and Development Institute for Gas Turbines COMOTI, 061126 Bucharest, Romania; raluca.rosu@comoti.ro (R.A.R.); oana.dumitrescu@comoti.ro (O.D.)

**Keywords:** passive morphing, biological mechanisms, bio-inspired aerospace structures

## Abstract

Passive morphing enables insect wings to adapt their shape under aerodynamic and inertial loads without direct actuation, contributing to efficient, stable, and agile flight. These natural mechanisms have attracted growing interest as a source of inspiration for adaptive aerospace structures. This review examines the structural and material features governing passive morphing, including wing geometry, venation architecture, membrane mechanics, and material heterogeneity, and discusses their influence on wing deformation and aerodynamic performance. Experimental approaches for characterizing wing kinematics and deformation, together with computational modelling techniques ranging from reduced-order models to fluid–structure interaction simulations, are comparatively evaluated, with emphasis on their capabilities, limitations, and validation requirements. The translation of biological morphing principles into engineering applications, including morphing wings, bio-inspired unmanned aerial vehicles, and lightweight adaptive structures, is also reviewed. Finally, the paper identifies current challenges and future research directions, emphasizing the need for integrated experimental and computational frameworks capable of bridging biological understanding with engineering design. By consolidating recent advances across biomechanics and aerospace engineering, this review provides a comprehensive perspective on passive morphing, emphasizing the transfer of biological mechanical principles rather than the direct scaling of insect-wing behaviour to aerospace applications.

## 1. Introduction

The pursuit of enhanced aerodynamic efficiency, operational flexibility, and reduced environmental impact has driven the continuous evolution of aerospace structures over the past decades. Conventional fixed-wing aircraft are generally designed to achieve optimal performance under a limited set of flight conditions, typically representing a compromise between conflicting mission requirements such as take-off, climb, cruise, maneuvering, and landing. Consequently, their aerodynamic configuration remains suboptimal during a significant portion of the flight envelope, leading to increased drag, reduced fuel efficiency, and limited adaptability to changing operational environments [1,2,3,4,5]. As modern aviation increasingly demands aircraft capable of performing multiple missions while maintaining high efficiency, there has been growing interest in adaptive structures that can continuously modify their aerodynamic characteristics in response to varying flight conditions rather than relying on discrete control surfaces alone [2,3,4,5,6].

Morphing technology has emerged as one of the most promising approaches for achieving adaptive aerodynamic performance. Unlike conventional control surfaces, which introduce abrupt geometric discontinuities and often increase drag, morphing structures seek to alter the overall geometry of aerodynamic lifting surfaces in a smooth and continuous manner. By modifying parameters such as wing camber, twist, span, sweep, chord, or even the entire wing planform, morphing aircraft can maintain favourable aerodynamic characteristics across a broad range of operating conditions while simultaneously improving maneuverability, stability, and energy efficiency [2,3,5]. Such adaptive capabilities have attracted considerable attention from both academia and industry, leading to extensive research programs investigating innovative structural concepts, compliant mechanisms, smart materials, and multidisciplinary optimization strategies for next-generation aircraft [3,5,6].

The concept of changing wing geometry is not entirely new. Early aviation pioneers recognized that modifying the wing shape could provide enhanced flight control long before the development of conventional ailerons. Advances in computational mechanics, lightweight composite materials, additive manufacturing, and smart actuators have renewed interest in morphing technologies, enabling concepts that were previously impractical to become increasingly feasible for modern aerospace applications [2,3,5,7].

**Figure 1:**
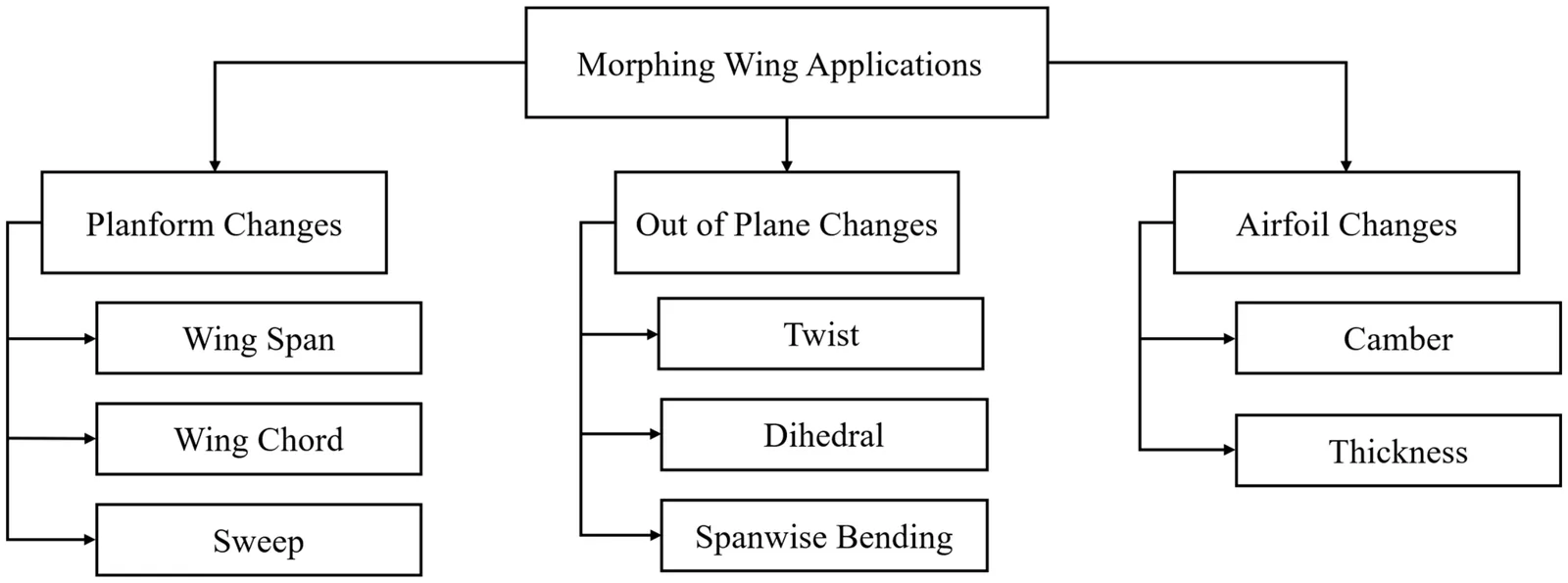
Classification of the principal morphing strategies employed in adaptive aircraft wings, including planform modifications, out-of-plane deformations, and airfoil shape changes [9].

Current morphing systems encompass a broad spectrum of structural solutions ranging from localized trailing-edge deformation to global modifications of wing geometry. Depending on the application, morphing may involve changes in airfoil camber, wing twist, span extension, sweep variation, folding mechanisms, or combinations of multiple deformation modes within a single structure [2,5,8]. These morphing strategies can be broadly classified into planform modifications, out-of-plane deformations, and airfoil shape changes, as illustrated in Figure 1. The successful implementation of these concepts requires the simultaneous satisfaction of several competing design requirements. Morphing structures must remain sufficiently stiff to withstand aerodynamic loads while also possessing adequate flexibility to undergo significant shape changes with minimal energy consumption. Achieving this balance represents one of the principal engineering challenges in adaptive aircraft design and requires close integration between aerodynamics, structural mechanics, materials science, and control engineering [5,7,8].

Existing morphing technologies can generally be classified into active and passive approaches according to the source responsible for generating structural deformation. Active morphing relies on dedicated actuation systems capable of producing controlled geometric changes in response to pilot commands or autonomous flight control algorithms. These systems commonly employ hydraulic actuators, electric motors, pneumatic devices, piezoelectric materials, or shape memory alloys to actively modify the structural configuration during flight [10,11]. Significant progress has been made in actively controlled morphing wings through the integration of smart materials capable of generating large recoverable strains while retaining structural functionality. Among these, shape memory alloys have received considerable attention due to their high actuation energy density, compact size, and ability to serve as both structural and actuation components, making them particularly suitable for variable-camber and twist applications [7]. Nevertheless, active morphing systems often introduce additional structural complexity, increased weight, higher manufacturing costs, and continuous power requirements that may partially offset their aerodynamic benefits [2,5,7].

In contrast, passive morphing exploits the intrinsic mechanical response of a structure to externally applied aerodynamic or inertial loads without requiring dedicated actuation. Instead of actively imposing deformation, passive systems are carefully designed so that their geometry evolves naturally under operational loading conditions. Structural flexibility, anisotropic stiffness distributions, compliant mechanisms, material heterogeneity, and aeroelastic coupling collectively determine the resulting deformation patterns, allowing the structure to continuously adapt to changing aerodynamic environments while consuming little or no additional energy [4,5,12]. Because deformation is governed primarily by the interaction between structural properties and external loads, passive morphing offers several advantages over actively actuated systems, including reduced mechanical complexity, lower maintenance requirements, improved reliability, and potentially lower structural weight. These characteristics make passive morphing particularly attractive for lightweight aerial vehicles operating under stringent mass and power constraints, such as micro air vehicles (MAVs), unmanned aerial vehicles (UAVs), and small-scale bio-inspired flying platforms [4,13].

Despite these advantages, designing effective passive morphing structures remains a highly complex multidisciplinary problem. Structural compliance must be carefully tailored to generate beneficial deformation while preventing excessive aeroelastic instabilities, fatigue accumulation, or structural failure. Furthermore, passive deformation cannot be directly prescribed during operation, requiring designers to accurately predict the coupled interaction between aerodynamic loading, structural response, and material behaviour across a wide range of flight conditions [5,14,15]. Consequently, modern morphing research increasingly combines experimental characterization, computational structural mechanics, fluid–structure interaction simulations, and optimization techniques to identify structural architectures capable of producing desirable adaptive responses while maintaining sufficient structural integrity throughout their operational lifetime [3,5,15].

The remarkable flight capabilities exhibited by insects provide one of the most compelling examples of passive structural adaptation found in nature. Unlike conventional engineered wings, insect wings operate as highly flexible composite structures whose deformation is not primarily dictated by active muscular control, but rather emerges from the complex interaction between aerodynamic loading, inertial forces, structural geometry, and heterogeneous material properties. During each flapping cycle, insect wings undergo continuous spanwise bending, chordwise bending, twisting, camber variation, and localized folding, allowing them to adapt their aerodynamic profile without the need for dedicated actuation mechanisms. These passive deformations contribute to lift enhancement, drag reduction, improved maneuverability, gust rejection, and energy-efficient flight, enabling insects to perform complex aerial maneuvers with remarkable stability despite their lightweight construction [11,12,13,15,16].

The exceptional performance of insect wings originates from a sophisticated structural organization that has evolved through millions of years of natural selection. Rather than behaving as homogeneous elastic membranes, insect wings consist of an intricate network of veins, flexible membranes, resilin-rich joints, and regions of spatially varying stiffness that collectively determine their mechanical response under loading. This hierarchical organization enables different regions of the wing to deform in a coordinated manner while maintaining sufficient structural integrity to withstand repeated cyclic loading. Consequently, wing deformation is neither entirely passive nor completely controlled, but instead results from a finely tuned balance between structural compliance and aerodynamic forcing. Understanding this balance has become a central topic in biomechanics, as it offers valuable insights into how complex structural adaptations can emerge from relatively simple passive mechanical principles [11,12].

The growing interest in bio-inspired engineering has consequently shifted attention toward translating these biological mechanisms into aerospace applications. Instead of reproducing insect wings directly, researchers seek to identify the underlying mechanical principles responsible for passive morphing and incorporate them into engineered adaptive structures. Such an approach has motivated the development of compliant wings, flexible skins, lattice architectures, multifunctional composites, and distributed stiffness concepts capable of reproducing some of the adaptive behaviour observed in natural flyers. Recent advances in additive manufacturing, topology optimization, and smart materials have further expanded the possibilities for manufacturing lightweight structures with spatially tailored mechanical properties, making biologically inspired passive morphing increasingly feasible for practical engineering applications [3,7,16,17].

Passive morphing has proven particularly attractive for micro air vehicles and unmanned aerial vehicles operating at low Reynolds numbers, where structural flexibility can significantly influence aerodynamic performance. Unlike large transport aircraft, whose structures are primarily designed to minimize deformation, small aerial vehicles often benefit from controlled flexibility that enables adaptive load redistribution, enhanced maneuverability, and improved robustness under unsteady atmospheric conditions. Several bio-inspired UAV concepts have therefore incorporated flexible wings, compliant mechanisms, or distributed stiffness variations inspired by insect flight in an effort to improve flight efficiency while simultaneously reducing system complexity and power consumption [11,12,17]. Although these developments demonstrate the considerable engineering potential of passive morphing, accurately reproducing the multifunctionality observed in biological systems remains an important challenge. Biological structures simultaneously satisfy aerodynamic, structural, energetic, and durability requirements through hierarchical architectures that are difficult to replicate using conventional engineering materials and manufacturing processes [3,5].

**Figure 2:**
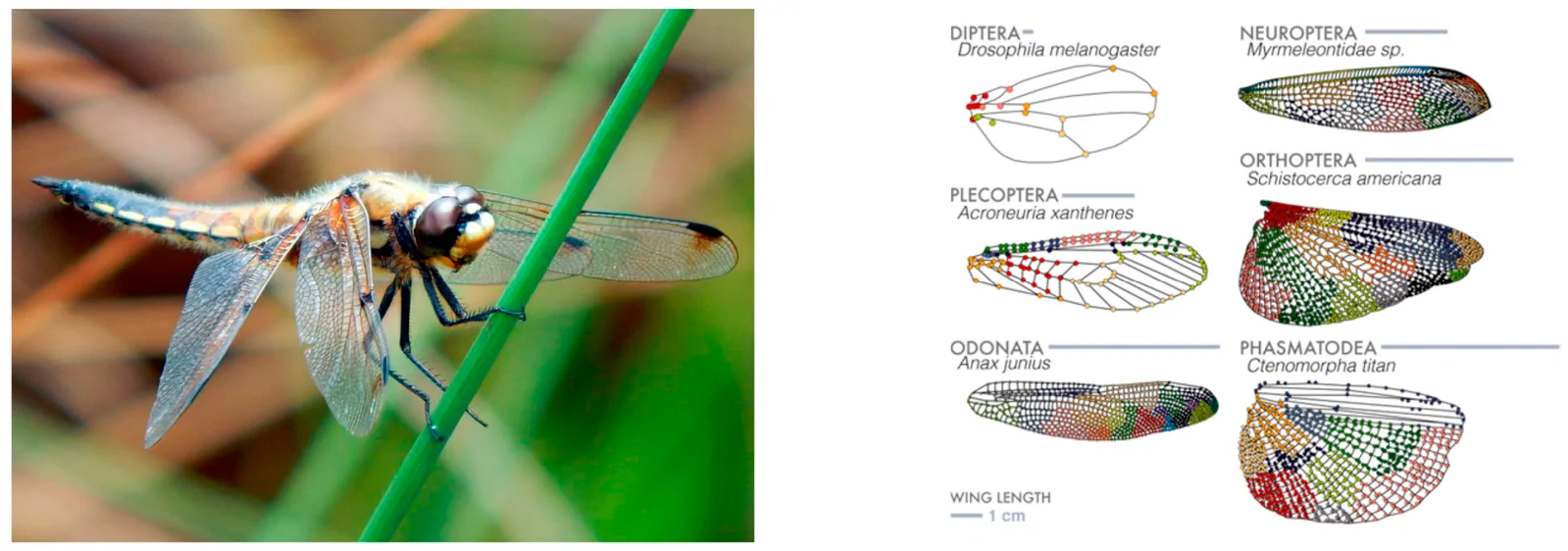
Diversity of insect-wing morphology and venation architecture. The dragonfly illustrates the lightweight membranous construction characteristic of insect wings, while the representative venation patterns from Diptera, Neuroptera, Plecoptera, Orthoptera, Odonata, and Phasmatodea demonstrate variations in wing geometry, vein density, and network organization among insect orders. These structural differences contribute to order-specific stiffness distributions and passive deformation responses during flight [18].

Among biological flyers, insects exhibit a particularly diverse range of wing architectures adapted to different aerodynamic and structural requirements. Although insect wings share a common organization consisting of a thin membrane reinforced by veins, their planform geometry, venation density, and vein connectivity vary considerably among taxonomic orders. As illustrated in Figure 2, these variations create distinct load-bearing networks that control stiffness distribution, structural anisotropy, and passive deformation during flight.

**Figure 3:**
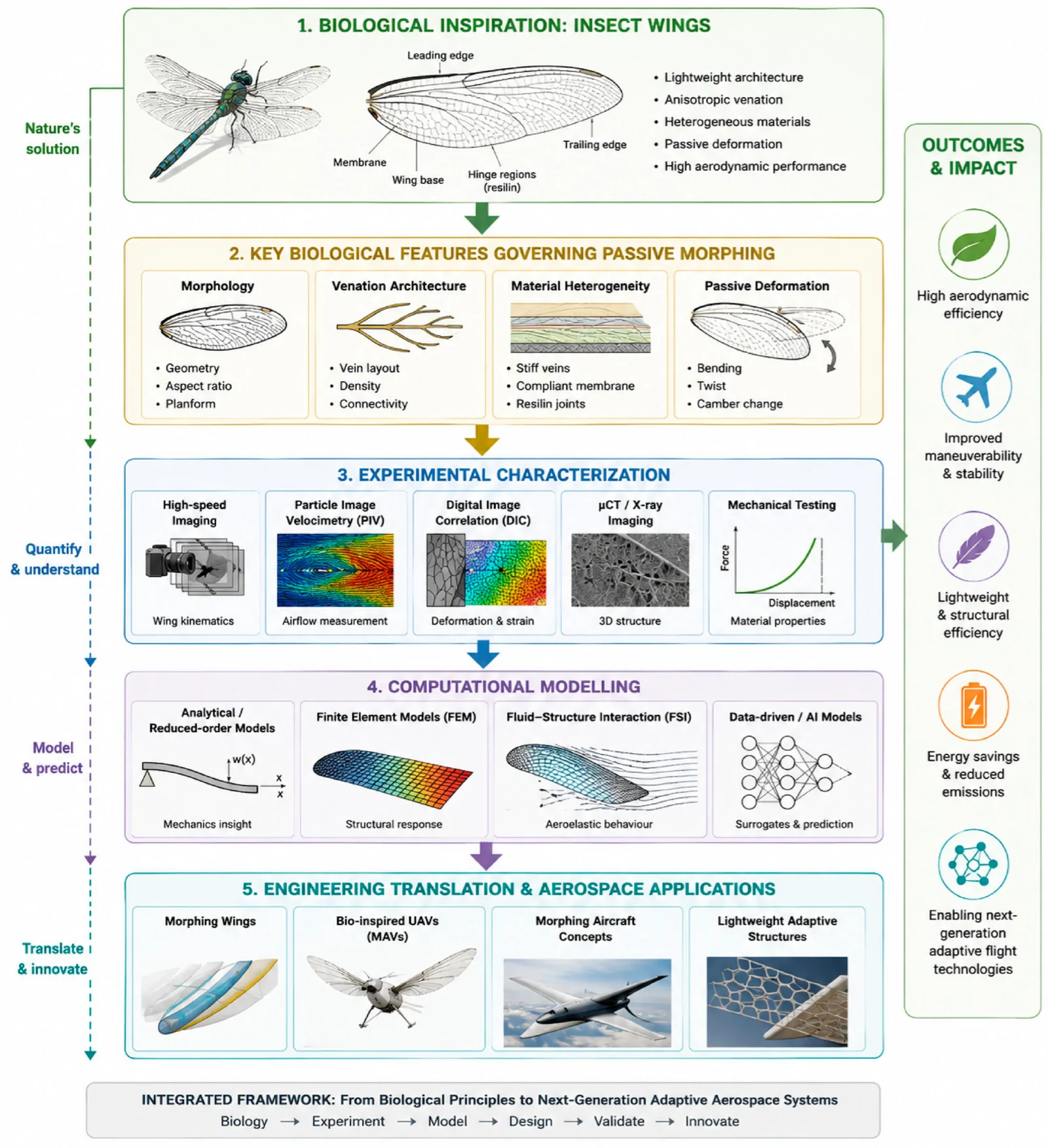
Conceptual framework of passive morphing research, illustrating the progression from biological inspiration through experimental characterization and computational modelling to bio-inspired aerospace applications.

Despite substantial progress in both insect biomechanics and morphing aircraft research, these two disciplines have largely evolved independently. Biological investigations have focused primarily on understanding wing morphology, material composition, deformation mechanisms, and flight performance, whereas aerospace research has concentrated on structural implementation, actuation strategies, manufacturing technologies, and aerodynamic optimization. As a result, relatively few studies have systematically examined how passive morphing mechanisms identified in insect wings can be translated into practical aerospace structures through integrated experimental and computational frameworks. Bridging this interdisciplinary gap requires not only a detailed understanding of biological deformation mechanisms but also an appreciation of the engineering constraints associated with structural scalability, manufacturability, reliability, and certification [3,5,9]. Figure 3 should be interpreted not only as a workflow, but as a synthesis of the principal relationships governing bio-inspired passive morphing. Across the reviewed studies, effective engineering translation consistently depends on three linked principles: controlled structural anisotropy, spatially distributed compliance, and coupling between aerodynamic loading and deformation. Experimental and computational methods provide complementary means of quantifying these relationships, while aerospace implementation requires their reformulation in terms of manufacturable stiffness distributions, compliant architectures, and validated aeroelastic response. The framework therefore emphasizes transfer of mechanical function rather than direct replication of biological morphology.

Unlike previous reviews that primarily address insect-inspired flapping flight, specific passive mechanisms, or morphing-aircraft technologies, this review focuses specifically on the translation of passive morphing principles from insect wings to aerospace structures. Its novelty lies in integrating biological morphology, venation architecture, material heterogeneity, and passive deformation with the experimental and computational methods required to quantify these mechanisms and their subsequent engineering implementation. Particular emphasis is placed on identifying transferable mechanical principles, evaluating the limitations that constrain their scalability and manufacturability, and defining the research gaps that must be addressed for their application in practical adaptive aerospace systems. This biology-to-engineering perspective is summarized in the integrated framework presented in Figure 3.

This review aims to establish such a connection by providing a comprehensive overview of passive morphing in insect wings and its relevance to adaptive aerospace structures. First, the biological mechanisms governing passive wing deformation are examined, with emphasis on wing morphology, venation architecture, membrane mechanics, material heterogeneity, and their influence on structural behaviour. Subsequently, the principal experimental techniques and computational approaches used to investigate insect wing deformation are critically discussed, highlighting both their capabilities and current limitations.

The remainder of this review is organized as follows: Section 2 examines the biological foundations of passive morphing in insect wings, focusing on wing architecture, material composition, structural heterogeneity, and passive deformation mechanisms. Section 3 reviews the principal experimental and computational approaches used to characterize wing deformation, aerodynamic interactions, and fluid–structure coupling, together with the strategies employed for model validation. Section 4 addresses the translation of these biological principles into aerospace engineering, including bio-inspired morphing concepts, structural and material solutions, and the principal challenges associated with scaling, manufacturing, control, and reliability. Finally, Section 5 identifies the remaining research gaps and emerging directions required to advance passive morphing toward more predictive, integrated, and practically deployable aerospace systems, before summarizing the principal conclusions of the review.

## 2. Biological Basis of Passive Morphing

Passive morphing in insect wings emerges from the intricate interaction between wing morphology, material heterogeneity, structural architecture, and aerodynamic loading. Rather than relying on active actuation, insects exploit spatial variations in geometry and mechanical properties to generate controlled deformations that continuously adapt the wing to changing flight conditions. Understanding these biological design principles is fundamental for identifying the mechanisms responsible for passive twist, bending, and camber formation, as well as for translating these strategies into bio-inspired aerospace structures. This chapter reviews the anatomical organization, material composition, deformation mechanisms, and functional significance of insect wings that collectively enable passive morphing.

### 2.1. Wing Morphology and Structural Organization

**Figure 4:**
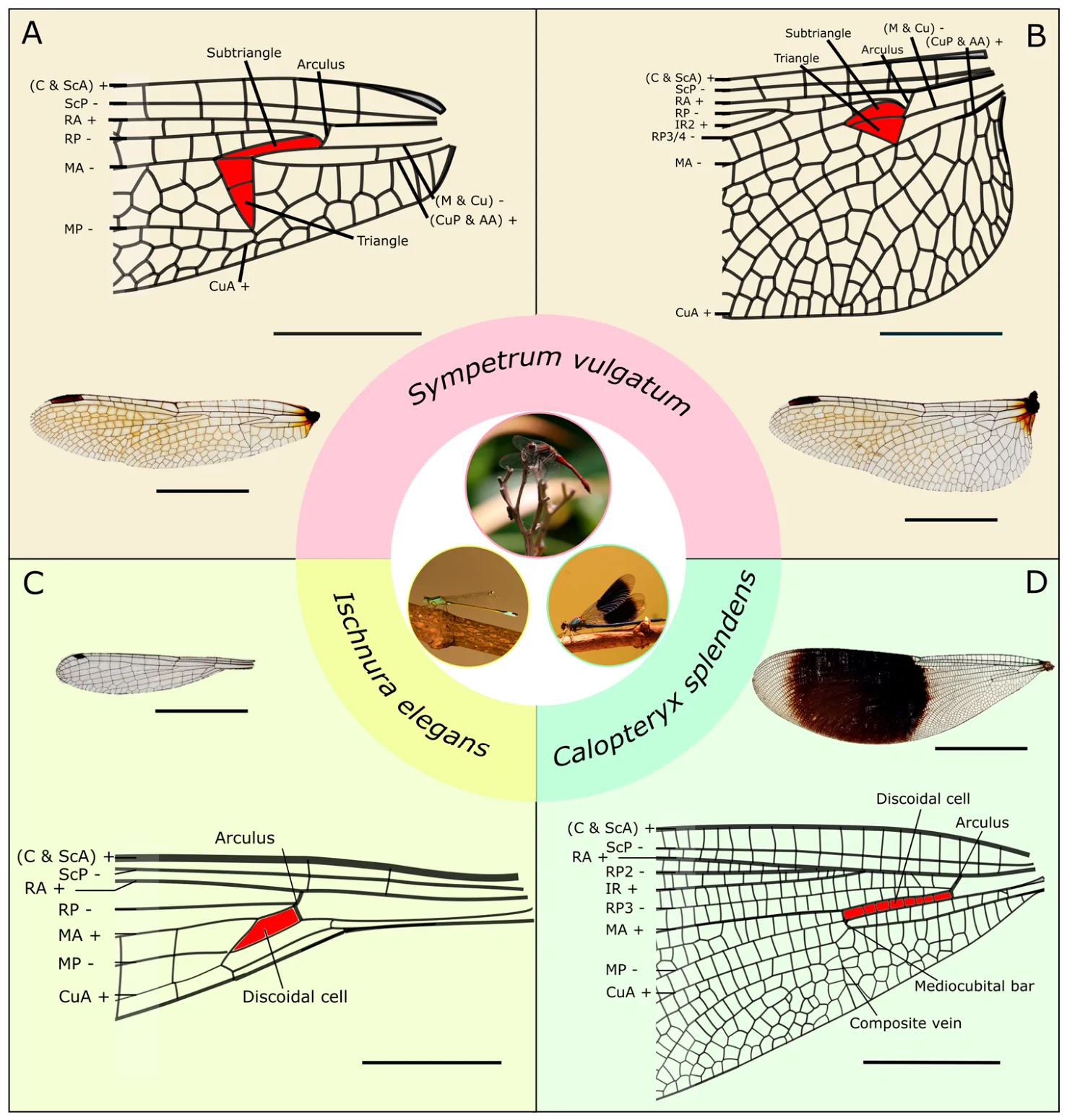
Representative diversity of insect wing morphology and venation architecture among Odonata species, illustrating variations in wing shape, vein organization, and specialized structural elements associated with different aerodynamic and biomechanical functions: dragonfly *S. vulgatum* (**A**) forewing; (**B**) hindwing; (**C**) damselfly *I. elegans* forewing; (**D**) damselfly *C. splendens* forewing [29].

The evolutionary origin and structural organization of insect wings have been reassessed through fossil evidence, biomechanical models, and developmental studies [19,20]. Paleozoic Pterygote fossils showing articulated, mobile winglets with functional venation challenge the classical paranotal theory and support a pleural/epicoxal origin in which mobility and articulation were ancestral features [19,20]. Insect wings subsequently diversified into a wide range of morphologies and venation patterns adapted to different flight strategies while retaining a common structural framework (Figure 4).

Wing planform and aspect ratio are key determinants of aerodynamic and structural performance, influencing lift generation, efficiency, and maneuverability during flapping and gliding flight [19,20]. Their interaction with unsteady aerodynamic phenomena, particularly leading-edge vortex (LEV) dynamics, further affects force generation [21,22].

The aerodynamic mechanisms underlying hovering and flapping insect flight were established through foundational theoretical and experimental studies, including the work of Weis-Fogh [23] and Ellington [24], which provided an important basis for subsequent investigations of unsteady aerodynamic phenomena such as leading-edge vortex formation, wing rotation, wake interactions, and clap-and-fling.

The venation architecture forms the primary load-bearing framework, providing structural stiffness while enabling controlled deformation [19,21]. Although vein development is governed by complex genetic and morphogenetic mechanisms [25,26,27], the resulting network geometry is particularly relevant from a mechanical perspective, as it contributes to an efficient balance between stiffness and structural mass [21,28]. Their interaction with unsteady aerodynamic phenomena, including leading-edge vortex (LEV) formation and evolution, can further influence force generation, depending on Reynolds number, wing geometry, and kinematics [21,22]. The ultrathin cuticular membrane spanning this network functions as a flexible load-transferring surface, transmitting aerodynamic loads while accommodating passive deformation during flapping [19,20,22,25]. These principal structural components and their functions are summarized in Table 1.

**Table 1:** Structural Organization of Insect Wing Morphology.

Morphological Element	Primary Biological & Biomechanical Function	Key Structural/Mechanical Determinants	References
**Planform**	Defines 2D area distribution; influences aerodynamic force generation and power expenditure.	Spatial area distribution relative to the rotation axis; spanwise distribution of wing area.	[19,20,21]
**Aspect Ratio (AR)**	Governs the trade-off between aerodynamic efficiency and maneuverability.	Ratio of wingspan to mean chord; interaction with leading-edge vortex stability.	[20,21,22]
**Venation**	Primary structural skeleton; provides flexural rigidity, anisotropic bending, and crack-arresting boundaries.	Vein topology, density and connectivity; longitudinal/cross-vein arrangement; local vein geometry and stiffness distribution.	[19,21,25,26,27,28]
**Membrane**	Acts as a flexible load-transferring surface, transferring aerodynamic loads to the venation network while permitting elastic deformation and camber.	Membrane thickness and elasticity; vein–membrane coupling; local pre-stress and corrugation.	[19,20,22,25]

### 2.2. Material Properties

The mechanical behaviour and dynamic response of insect wings during flight are determined by the interaction between material composition, structural geometry, and local material specialization [30]. Resilin is an important component of compliant regions within insect wings and is characterized by high elasticity and elastic recovery [31,32]. In dragonfly wings, Appel et al. [33] identified resilin within vein joints and demonstrated that vein walls possess a heterogeneous composite organization in which resilin-containing regions are integrated with more strongly sclerotized cuticle. These compliant regions allow local deformation while the surrounding cuticular structures maintain the load-bearing function of the venation network [32,33]. Cuticular spikes and other joint geometries can additionally restrict deformation in one direction, producing mechanically asymmetric joints that combine compliance with protection against excessive deformation [33].

The mechanical response of the venation network is governed not only by material properties but also by vein cross-sectional geometry and spatial organization. Wing veins form hollow tubular structural elements whose dimensions and material composition vary along the wing [32,33]. Ultrastructural observations by Appel et al. [33] demonstrated substantial variation in vein-wall organization and thickness, together with spatial variation in the distribution of resilin and sclerotized cuticle. These observations support treating insect-wing veins as structurally heterogeneous composite elements rather than homogeneous beams. Such geometric and material heterogeneity contributes to the strongly direction-dependent bending and torsional behaviour of insect wings [30,33].

The membrane spanning the venation network provides a lightweight surface through which aerodynamic loading is transferred to the supporting veins [30,34]. Its mechanical behaviour cannot be considered independently of the surrounding venation. Dirks and Taylor [34], for example, experimentally demonstrated that veins substantially increase the fracture resistance of insect wings by arresting or deflecting propagating cracks. Thus, the mechanical performance of the wing emerges from the combined membrane–vein architecture rather than from the membrane material alone.

Material heterogeneity also occurs across multiple structural scales. Appel et al. [33] showed that dragonfly wing veins contain distinct cuticular layers and resilin-containing regions, producing local transitions between comparatively stiff and compliant material. At the whole-wing scale, mechanical measurements by Combes [35,36] demonstrated pronounced spatial and directional variation in flexural stiffness associated with venation and wing geometry. These stiffness distributions are important because they determine where deformation is concentrated when the wing is subjected to aerodynamic and inertial loading. At a smaller structural scale, Rajabi et al. [37] demonstrated that asymmetric double-layer membrane structures can act as direction-dependent compliant hinges, providing substantially different mechanical responses according to the direction of loading. Together, these observations show that passive morphing depends on the coordinated distribution of geometry, material composition, and local compliance rather than on a single material property. Table 2 summarizes the mechanical properties of the principal structural materials found in insect wings.

### 2.3. Passive Deformation Mechanisms

Insect wings function as complex aeroelastic structures that undergo passive twist, camber, and bending in response to aerodynamic and inertial loading [36,38]. Rather than being determined by flexibility alone, these deformation modes depend on the spatial distribution of flexural and torsional stiffness, wing geometry, and venation architecture [30,35,36]. Twist modifies the local orientation of wing sections during the flapping cycle, while chordwise deformation contributes to changes in camber and local aerodynamic geometry [35,36,37,38,39,40].

The importance of structural anisotropy has been demonstrated experimentally by Combes and Daniel [35,36], who measured flexural stiffness in insect wings and showed that stiffness varies substantially with bending direction and position along the wing. This anisotropy allows the venation network to provide sufficient spanwise support while retaining greater compliance in directions where passive deformation is required. Consequently, deformation is spatially distributed rather than occurring uniformly across the wing [35,36].

Localized structural features further regulate this response. Resilin-containing vein joints provide compliant regions within an otherwise more strongly sclerotized venation network [33], while asymmetric membrane and hinge architectures can produce direction-dependent deformation. Rajabi et al. [37], for example, demonstrated an asymmetric double-layer membrane hinge whose mechanical response differed according to the direction of bending. Such structural asymmetry provides a mechanism through which deformation can be mechanically constrained without active control.

Passive deformation is also exploited for larger structural transformations. Wing folding in insects can involve geometrically programmed folding lines, elastic joints, and mechanically stable configurations that permit compact storage and subsequent deployment [31,38,41]. Dynamic modelling further indicates that the interaction between structural properties, wing inertia, and imposed flapping motion determines the resulting deformation and vibration response [38]. Overall, passive wing morphing emerges from the interaction of venation geometry, material heterogeneity, structural anisotropy, and localized compliance rather than from any individual structural component [33,37,38,39,40,41]. Table 3 summarizes the mechanical deformation mechanisms associated with insect wing structures.

### 2.4. Functional Significance

The functional design of insect wings reflects long-term evolutionary optimization, with wing morphology and material properties adapting to ecological demands. Wing aspect ratio and loading strongly influence flight efficiency and metabolic cost, with high-aspect-ratio wings favoring long-distance migration through reduced induced drag, while specialized wing planforms support the high-lift flapping required for rapid maneuvering in predation and territorial defense [42]. The wing’s structural integration—specifically the use of resilin-rich joints and fiber-composite venation—enables efficient storage and release of elastic energy, further reducing the overall metabolic burden of flight [22,39]. Stability in flight is a critical requirement for maintaining controlled trajectories in turbulent environments, such as forest canopies or high-velocity airflows [42,43]. Structural features such as the pterostigma function as passive inertial dampers that reduce buffeting and high-frequency vibrations, while the wing’s three-dimensional corrugated structure acts as a rigid truss to maintain aerodynamic stability during high-frequency flapping [22]. This passive stabilization is essential for maintaining the wing’s angle of attack against fluid–structure instabilities, ensuring consistent force production [22,43]. Maneuverability is largely governed by the wing’s ability to undergo rapid, asymmetric passive deformations [41,42]. By exploiting the relationship between wing planform, centre-of-mass position, and second moment of area, insects achieve high rotational agility, enabling complex maneuvers such as rapid banking turns and vertical escape trajectories through highly responsive wing torsion [42]. Innovations in bio-inspired robotics have demonstrated that artificial wings incorporating local stiffness gradients and specialized coupling mechanisms can replicate this high level of flight control and maneuverability observed in nature [21,41,44]. Finally, load alleviation serves as a vital survival mechanism, allowing insects to withstand high inertial stresses during rapid maneuvers or collisions without permanent structural damage [42,43]. The hierarchical architecture of wing veins, characterized by a “hard-shell, soft-core” sandwich design, effectively redistributes peak stresses, moves tensile stresses toward the surface, and arrests the propagation of fatigue cracks [22,43]. Furthermore, the passive buckling of wing segments under excessive load acts as a protective mechanism, preventing catastrophic structural collapse [42,43]. These load-alleviating strategies are of profound interest for aerospace engineering, where mimicking these natural, fail-safe architectures can optimize the stiffness-to-weight ratio of airfoils and extend the operational life of lightweight micro-air vehicles [22,43].

## 3. Experimental and Computational Investigation

Understanding passive morphing in insect wings requires the integration of experimental observations with computational modeling, as neither approach alone can fully characterize the complex aeroelastic interactions governing wing deformation. Experimental techniques provide quantitative measurements of wing kinematics, structural deformation, internal morphology, and aerodynamic flow fields, while computational models enable detailed analysis of structural behavior, fluid dynamics, and fluid–structure interactions under controlled conditions. Together, these complementary approaches offer a comprehensive framework for investigating the mechanisms underlying passive morphing and for translating biological design principles into bio-inspired engineering applications. This chapter reviews the principal experimental methodologies, computational modeling strategies, and validation approaches currently employed to study passive wing morphing in insects.

### 3.1. Experimental Techniques

Experimental investigation of passive morphing in insect wings presents significant technical challenges owing to the small size of insect wings, their high flapping frequencies, rapid structural deformations, and the strong coupling between aerodynamic loading and structural response. Because passive morphing is governed by complex aeroelastic interactions occurring over millisecond timescales, modern experimental studies increasingly combine multiple complementary measurement techniques to simultaneously characterize wing kinematics, structural deformation, force generation, and surrounding flow structures [44,45,46,47,48].

High-speed videography represents the cornerstone of experimental insect flight research for quantifying wing kinematics, with modern systems operating at several thousand frames per second to resolve the complete wingbeat cycle even for frequencies exceeding 200 Hz. Multiple synchronized cameras enable the three-dimensional reconstruction of wing motion, allowing researchers to quantify wingtip trajectories, instantaneous angles of attack, wing rotation during pronation and supination, stroke plane orientation, and spanwise deformation throughout the flapping cycle [48,49,50,51]. These measurements demonstrate that insect wings rarely behave as rigid bodies; instead, passive deformation continuously modifies local wing geometry during both downstroke and upstroke to directly influence aerodynamic forces [46,47], while also providing essential realistic boundary conditions for computational fluid dynamics (CFD) and finite element (FE) structural analyses [47]. Building upon basic videography, quantitative motion tracking replaced manual digitization by using algorithms to automatically reconstruct body orientation and wing kinematics throughout the entire flapping cycle, significantly reducing operator bias [52,53].

**Figure 5:**
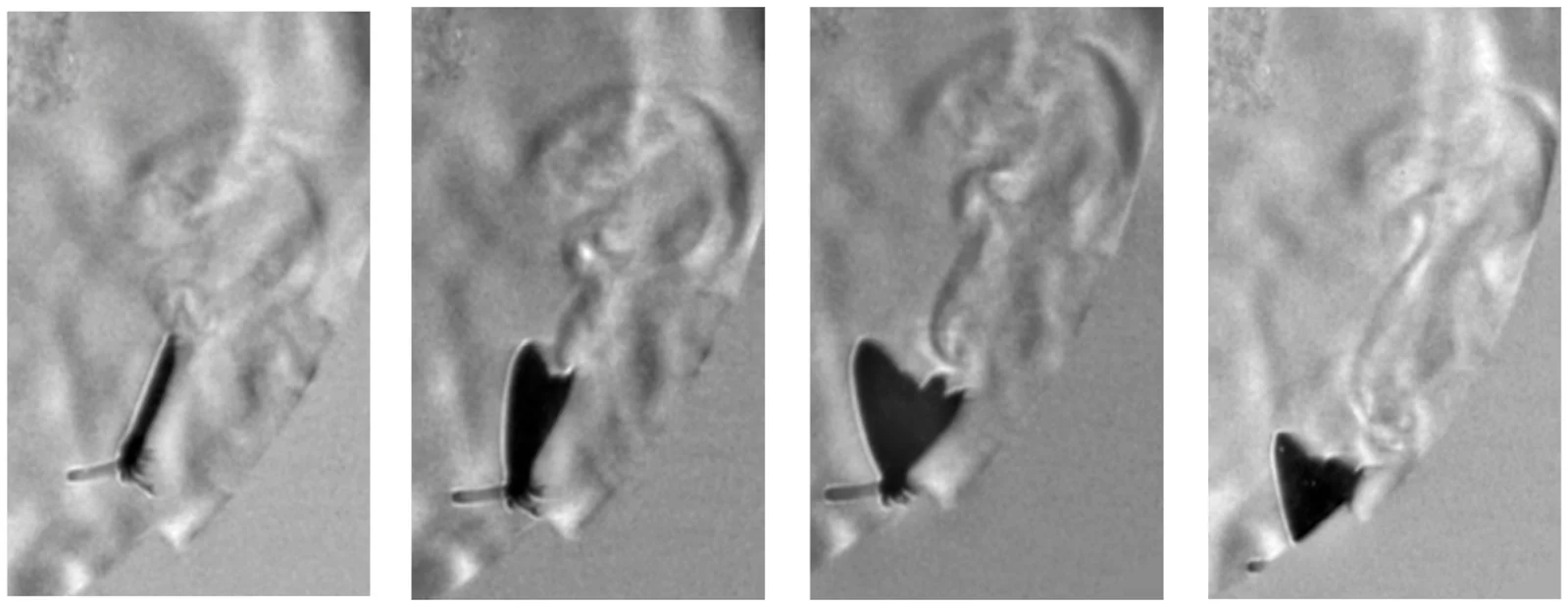
Post- processed schlieren images of an unsteady flow field generated by a flapping wing of a butterfly, showing the formation, shedding, and downstream evolution of coherent vortical structures during successive phases of the flapping cycle [56].

Multi-camera reconstruction, hull methods, principal component analysis, and model-based tracking now enable complete 3D recovery of wing kinematics and passive deformation, even during overlap or partial occlusion. Sub-pixel accuracy is essential because small changes in spanwise twist or camber can alter local angles of attack and significantly affect lift and vortex formation [49,51]. While high-speed imaging details wing motion, flow visualization techniques are required to directly observe the surrounding flow field, unsteady aerodynamic mechanisms, vortex formation, and wake evolution. Early qualitative methods utilizing smoke streams or dye injection demonstrated that insects exploit unsteady flow structures rather than quasi-steady lift [44,45], which was later transformed by Particle Image Velocimetry (PIV) and Digital Particle Image Velocimetry (DPIV) to enable quantitative measurements of instantaneous velocity fields by seeding tracer particles illuminated by laser sheets [54,55]. DPIV studies have provided quantitative characterization of leading-edge vortex (LEV) formation and evolution and have shown that, under certain experimental conditions, the LEV can remain attached during substantial portions of the translational stroke, contributing to enhanced aerodynamic force generation. However, LEV development, attachment, and shedding depend strongly on Reynolds number, wing geometry, kinematics, and the specific experimental configuration [44,56]. These studies have also characterized other unsteady aerodynamic mechanisms, including rotational circulation, wake capture, and clap-and-fling interactions. Complementing PIV, schlieren and shadowgraph techniques visualize density gradients and wake evolution without introducing tracer particles, offering qualitative insights over large spatial domains when synchronized with high-speed videography [56], while direct aerodynamic force measurements using sensitive load cells on tethered insects or robotic models remain essential for separating inertial and aerodynamic forces and validating observed kinematics and flow structures [44,45,47,55]. As illustrated in Figure 5, post-processed schlieren images provide a direct qualitative representation of the unsteady flow structures generated during wing motion. The successive frames reveal the formation, shedding, and downstream convection of coherent vortical structures, as well as their interaction with the wake produced during earlier phases of the motion. Although Schlieren visualization does not directly provide quantitative velocity or pressure fields, it captures the spatial and temporal evolution of density gradients over a large field of view, making it particularly valuable for identifying vortex trajectories, wake interactions, and cycle-to-cycle flow variations.

Digital Image Correlation (DIC) tracks naturally occurring surface features or artificial speckle patterns between successive images to measure local displacement, strain, curvature, and full-field deformation with sub-pixel accuracy [55,56,57]. Stereo-DIC overcomes the depth limitations of two-dimensional imaging by combining synchronized camera pairs to reconstruct three-dimensional out-of-plane deformation, while dual stereo-DIC configurations with polarized illumination and fluorescent speckles enable simultaneous measurement of both wings during rapid overlap [58], supplying high-fidelity deformation maps to calibrate material properties and validate finite element models [55,56,57].

Investigating the internal structural architecture and hierarchical organization of veins, membranes, and joints requires volumetric imaging, with X-ray micro-computed tomography (μCT) serving as the preferred non-destructive method to reconstruct intact wings with micrometre-scale accuracy [59]. Optimized preparation and staining procedures, such as using phosphotungstic acid (PTA), significantly enhance image contrast for dry or stained samples to clearly identify vein origins, membrane interfaces, and concealed articulation structures at the wing base [59,60], allowing high-resolution datasets to convert directly into surface meshes for finite element and computational fluid dynamics models [61,62]. This is further complemented by Scanning Electron Microscopy (SEM) for examining membrane corrugation and cuticular microstructure at high spatial resolution, and Confocal Laser Scanning Microscopy (CLSM) for identifying resilin-rich compliant joints via autofluorescence, demonstrating through multimodal approaches that passive morphing relies on the complex interplay between morphology, material composition, and structural organization [63].

Finally, the massive volume of data generated by these techniques has stimulated automated image analysis and morphometric reconstruction, replacing labor-intensive manual digitization [53] with advanced computer vision algorithms that segment wing membranes, extract venation networks, and identify vein junctions [64,65]. Specialized software platforms like WingGram automate the transition from biological images to computationally efficient finite element meshes while preserving realistic venation topology [65], and integrated platforms like WingAnalogy incorporate Procrustes alignment, asymmetry analysis, and statistical comparison to facilitate large-scale quantitative studies across species [65], collectively ensuring that high-speed imaging, motion tracking, optical flow diagnostics, DIC, volumetric imaging, advanced microscopy, and automated analysis seamlessly link experimental biology with numerical simulations [45,46,47,48,49,50,51,52,53,54,55,56,57,58,59,60,61,62,63,64,65]. The experimental techniques most frequently employed to characterize passive morphing are compared in Table 4.

### 3.2. Computational Models

Computational modelling provides a means of isolating the structural and aerodynamic mechanisms governing passive wing deformation under controlled conditions. However, the conclusions obtained from different modelling approaches are not directly interchangeable because the predicted response depends on the adopted geometry, structural idealization, material properties, Reynolds number, prescribed kinematics, and fluid–structure coupling strategy. Analytical, finite-element, fluid–structure interaction, reduced-order, and data-driven methods therefore provide complementary rather than equivalent descriptions of passive morphing.

Analytical models are useful when the objective is to identify dominant deformation mechanisms with limited computational cost. Kang and Shyy [66], for example, developed an analytical formulation coupling instantaneous aerodynamic loading and structural deformation for an insect-scale flexible wing in hover. Such formulations are particularly valuable for parametric studies because structural and aerodynamic parameters can be varied efficiently, but their predictive capability depends on simplifying assumptions regarding wing geometry, deformation modes, and aerodynamic representation. They therefore provide a mechanistic reference against which higher-fidelity numerical approaches can be interpreted rather than a complete representation of the three-dimensional aeroelastic problem.

Finite-element studies illustrate the influence of structural representation particularly clearly. Jin et al. [67] constructed an FEM model directly from a beetle wing approximately 50 mm in span and 20 mm in chord. The geometry was divided into 48 regions to represent spatial variations in membrane and vein thickness, and the predicted bending response was compared with experiments. In contrast, Darvizeh et al. [68] examined the free vibration of a dragonfly forewing using a three-dimensional model in which veins were represented as tubular elements with an outer diameter of 0.135 mm and a wall thickness of 0.025 mm, while the membrane was represented by 0.003 mm shell elements. Both veins and membrane were assigned an isotropic Young’s modulus of 7.5 GPa, a Poisson ratio of 0.3, and a density of 1200 kg m^−3^. Their calculated first four natural frequencies were 181.12, 313.28, 390.14, and 446.31 Hz, respectively, with bending dominating the fundamental mode. The measured flapping frequency considered in that study was approximately 27 Hz, corresponding to only about 15% of the first natural frequency and a calculated dynamic magnification factor of approximately 1.02.

These FEM results should not be interpreted as directly equivalent. Jin et al. [67] emphasized geometric and thickness heterogeneity and validated static bending behaviour, whereas Darvizeh et al. [68] focused on modal behaviour and represented the wing material as homogeneous and isotropic. The agreement reported within each study therefore applies to different physical quantities and structural assumptions. This comparison illustrates why apparently similar FEM approaches can lead to different levels of structural fidelity and why validation should be reported together with geometry, material model, loading condition, and measured quantity.

The influence of modelling assumptions becomes even more pronounced when aerodynamic loading is coupled to structural deformation. Fitzgerald et al. [69] compared two fluid–structure formulations based respectively on direct numerical simulation and an unsteady vortex-lattice representation. The lower-order vortex model reproduced many of the system-level trends while requiring considerably less computational effort, but discrepancies arose where viscous flow effects became important. This comparison demonstrates that reduced aerodynamic fidelity may be adequate for some design-level predictions but should not be assumed to reproduce all vortex dynamics resolved by viscous simulations.

Ishihara [70] subsequently used a strongly coupled FSI formulation to design an insect-inspired microflapping wing whose deformation was generated passively through structural–fluid interaction. For a driving frequency of 428 Hz, resonance amplified the wing tip displacement to approximately 19 times the base displacement. The resulting quasi-steady stroke amplitude reached approximately 73°, while passive pitching reached approximately 62°, values comparable to those targeted from insect flight. These results demonstrate that structural resonance and fluid–structure coupling can be exploited intentionally to generate large wing motions without prescribing the complete deformation kinematics.

A later FSI study by Ishihara and Onishi [71] examined passive feathering and cambering using a structural representation explicitly incorporating insect-wing characteristics. For one dynamically similar case, the nondimensional conditions were (Re = 819.8), (Ca = 0.2420), and a solid-to-fluid mass ratio of 0.001916. Parametric analysis showed that a representative wing configuration produced a tip feathering angle of approximately 31° and an average camber of 5.4%. The study further quantified the role of structural inertia: when the inertial contribution was varied, wing inertia accounted for approximately 11% of the cycle-averaged feathering response and 15% of the cycle-averaged camber response, with aerodynamic and elastic forces providing the dominant balance.

The comparison between the two Ishihara studies is especially informative. Both indicate that passive deformation can emerge from the coupled action of structural elasticity, inertia, and aerodynamic loading, but the magnitude and interpretation of the deformation depend on the structural scale and dynamic-similarity parameters. The 2022 study [70] emphasizes resonance-assisted micro-wing design at a high actuation frequency, whereas the later framework [71] isolates the relative contributions of aerodynamic, elastic, and inertial effects under dynamically scaled insect-flight conditions. Consequently, their results support a common passive-coupling mechanism but should not be reduced to a universal statement that flexibility necessarily improves aerodynamic performance.

A similar dependence on operating conditions is evident in aerodynamic simulations of rigid or prescribed-kinematic flapping wings. Ramamurti and Sandberg [72] reproduced the dynamically scaled *Drosophila* configuration of Dickinson et al. at approximately (Re = 136), using a wing with a 25 cm span and a mean chord of 6.7 cm in the scaled apparatus. Their computations showed that the timing of wing rotation relative to stroke reversal strongly altered the instantaneous force history: advanced rotation increased the thrust peak relative to symmetrical rotation, whereas delayed rotation reduced it. Thus, even for identical planform geometry and Reynolds number, differences in prescribed rotational timing change the aerodynamic response substantially.

Ansari et al. [73] reached broadly consistent conclusions regarding the importance of unsteady kinematics, but using a nonlinear vortex-based analytical model at Reynolds numbers of order 200. Comparison with Robofly data yielded mean lift discrepancies of 13% over the complete simulated sequence and 6.8% over the final cycle; corresponding thrust discrepancies were 7.2% and 3.3%, respectively. The experimental and predicted mean lift over the final cycle were 0.4081 and 0.3805 N, while the corresponding thrust magnitudes were 0.6106 and 0.5904 N. These results show that a reduced-order unsteady model can reproduce force histories reasonably closely under calibrated hovering conditions, while still underpredicting or overpredicting particular integrated quantities.

At the considerably larger MAV-scale Reynolds number of 25,000, Lankford et al. [74] used three-dimensional unsteady CFD to reproduce a rigid wing flapping at 5 Hz through a 107° stroke and translational pitch angles of 40°, 50°, and 60°. The predicted instantaneous forces generally remained within the experimental uncertainty, but the results were highly sensitive to pitch angle. The 50° case produced a maximum translational lift-to-drag ratio of approximately 0.75 and a power loading of 0.19 N W^−1^, compared with 0.16 and 0.14 N W^−1^ for the 40° and 60° cases, respectively. The 40° case also showed a noticeably larger discrepancy between experimental and computational power loading. These differences were attributed principally to the altered wing-pitch kinematics and associated LEV evolution.

Taken together, these primary studies show that agreement across computational approaches is conditional rather than universal. FEM predictions depend strongly on geometric and material idealization; FSI predictions depend on stiffness, mass ratio, resonance, and coupling assumptions; and aerodynamic force predictions depend strongly on Reynolds number and prescribed rotational or pitching kinematics. Quantitative comparisons therefore provide more useful evidence than assigning computational methods generic accuracy or cost categories. Representative numerical conditions and outcomes are summarized in Table 5.

### 3.3. Validation Strategies

The increasing complexity of computational models has significantly improved the prediction of insect wing aerodynamics and structural deformation. However, regardless of the numerical approach employed, computational results must be validated against experimental observations to ensure that the underlying physical mechanisms are accurately reproduced. Validation therefore represents an essential step in the development of analytical models, finite element simulations, fluid–structure interaction (FSI) frameworks, and reduced-order models. Rather than comparing only global aerodynamic quantities, recent studies increasingly evaluate multiple physical variables simultaneously, including wing kinematics, structural deformation, vortex evolution, and aerodynamic force generation.

Experimental validation generally combines several complementary measurement techniques to assess different aspects of wing performance. High-speed videography provides detailed information on wing kinematics and passive deformation throughout the flapping cycle, while Particle Image Velocimetry (PIV) enables quantitative comparison of simulated and measured flow fields, including leading-edge vortex (LEV) formation, wake evolution, and velocity distributions. These measurements are frequently complemented by direct force measurements, allowing lift, drag, and pitching moments predicted by numerical simulations to be compared with experimental results. By combining structural, aerodynamic, and kinematic measurements, researchers can evaluate not only the overall performance of a computational model but also its ability to reproduce the physical mechanisms responsible for passive morphing [55,58].

Analytical and reduced-order models are commonly validated by comparing predicted aerodynamic forces and wing kinematics with experimental measurements. Ansari et al. developed a nonlinear analytical model for hovering insect-like wings and validated the predicted force histories against published experimental data. Their results demonstrated that simplified vortex-based formulations can accurately reproduce the dominant unsteady aerodynamic mechanisms, including leading-edge vortex development and wake interactions, while maintaining significantly lower computational cost than high-fidelity numerical simulations [72].

Validation becomes increasingly comprehensive for Computational Fluid Dynamics (CFD) models. Ramamurti and Sandberg developed a three-dimensional CFD solver for flapping insect wings and compared the predicted aerodynamic forces with the experimental measurements reported by Dickinson et al. In addition to evaluating lift and drag histories, the authors performed grid-independence analyses and verified that the numerical model accurately reproduced the principal vortex structures responsible for aerodynamic force generation. This study demonstrated that reliable CFD simulations require validation of both integrated aerodynamic loads and the underlying flow physics [73].

Recent investigations increasingly combine multiple experimental datasets to validate numerical predictions. Lankford et al. assessed a three-dimensional CFD model of a bio-inspired flapping wing using both instantaneous force measurements and Particle Image Velocimetry (PIV). The numerical simulations showed good agreement with the measured aerodynamic forces while simultaneously reproducing the temporal evolution of the leading-edge vortex and its shedding throughout the wingbeat cycle. Such combined validation approaches provide greater confidence than force measurements alone because they verify both aerodynamic performance and the flow structures responsible for force generation [74].

For flexible wings, validation extends beyond aerodynamic loads to include structural deformation. High-speed imaging, Digital Image Correlation (DIC), and micro-computed tomography (μCT) are increasingly incorporated into validation workflows by providing realistic wing geometries, deformation fields, and material distributions for numerical models [58,59,60,61,62,63,64,65]. Experimental deformation measurements obtained using DIC can be directly compared with finite element predictions, while μCT-derived geometries improve the geometric fidelity of computational meshes. These complementary techniques reduce uncertainties associated with simplified wing geometries and material assumptions, leading to more accurate structural simulations [63,64,65,66,67,68].

Validation has also evolved from isolated wing analyses toward complete flapping-wing systems. Dong et al. [77] presented a coupled Computational Fluid Dynamics–Computational Structural Dynamics (CFD–CSD) framework incorporating flexible multibody dynamics and validated the numerical predictions against free-flight measurements of a flapping-wing micro air vehicle. Rather than considering only aerodynamic forces, the validation included body motion, wing deformation, and flight dynamics, demonstrating that integrated aeroelastic simulations can successfully reproduce realistic flapping flight and providing an important step toward the development of bio-inspired aerial vehicles.

Overall, validation strategies have evolved from simple comparisons of average aerodynamic forces toward comprehensive multi-physics assessments integrating wing kinematics, structural deformation, aerodynamic loading, and flow visualization. This combination of complementary experimental techniques enables more rigorous evaluation of computational models and increases confidence that the numerical predictions accurately capture the aeroelastic mechanisms governing passive morphing. Such integrated validation frameworks are becoming increasingly important as computational models continue to incorporate higher levels of structural and aerodynamic complexity. The relationship between experimental measurements and computational model validation is summarized in Table 6.

Despite increasing methodological sophistication, direct comparison among studies remains difficult because experimental and numerical investigations frequently employ different species, geometries, boundary conditions, kinematics, material assumptions, and validation metrics. Consequently, apparent differences in predicted deformation or aerodynamic performance may reflect methodological choices rather than genuine physical discrepancies. Simplified models provide useful mechanistic insight but may neglect nonlinear aeroelastic effects, whereas high-fidelity FSI offers greater physical completeness at substantially higher computational cost. Experimental techniques present similar trade-offs between temporal resolution, spatial resolution, dimensionality, and invasiveness. No single methodology therefore provides a complete description of passive morphing; the most reliable conclusions emerge when structural deformation, aerodynamic forces, and flow fields are evaluated using complementary experimental and computational approaches.

Across the reviewed literature, the strongest evidence concerns the existence of passive bending, twisting, and camber deformation and their dependence on wing architecture and distributed stiffness, as these mechanisms have been observed using multiple experimental and computational approaches. In contrast, quantitative conclusions regarding the aerodynamic benefit of specific deformation modes are less consistent, since reported effects depend strongly on species, Reynolds number, prescribed kinematics, structural boundary conditions, and material assumptions. Evidence for the contribution of individual material or geometric features is also more limited, as several conclusions originate from species-specific experiments or simplified numerical models rather than systematic cross-species validation. Apparent discrepancies between studies should therefore not necessarily be interpreted as contradictory physical mechanisms, but often reflect differences in experimental configuration and modelling fidelity. This heterogeneity currently prevents universal quantitative design rules and reinforces the need for standardized datasets and coupled experimental–computational validation.

## 4. Translation to Aerospace Engineering

In this review, an engineering morphing mechanism is classified as passive only when the deformation is generated by aerodynamic, inertial, or other external loading acting on the structural architecture, without dedicated actuation. Systems in which actuators or smart materials actively impose the deformation are classified as active, while concepts combining load-induced deformation with limited sensing or actuation are considered hybrid. Active and hybrid concepts are discussed in this section only where they provide relevant comparisons or potential pathways for implementing or controlling biologically inspired passive mechanisms.

### 4.1. Morphing Aircraft Structures

The development of morphing aircraft is driven by the limitations of conventional fixed-geometry wings, which require compromises between different flight conditions. Morphing structures overcome this limitation by enabling controlled changes in wing geometry during flight, allowing adaptation to varying speeds, altitudes, and mission profiles while improving aerodynamic efficiency. Unlike conventional hinged control surfaces, morphing wings aim to achieve smooth and continuous shape changes, with modifications to parameters such as span, sweep, chord, camber, twist, or thickness. By maintaining surface continuity and reducing geometric discontinuities, morphing can improve aerodynamic performance across a broader flight envelope [78,79,80].

Among the various morphing concepts, passive morphing has attracted increasing attention because it exploits the interaction between aerodynamic loading and structural flexibility rather than relying exclusively on active actuation systems. Similar to insect wings, passive morphing seeks to embed the desired deformation directly into the structural architecture by tailoring stiffness distribution and material anisotropy. As aerodynamic loads change during flight, the wing deforms naturally toward more favourable aerodynamic configurations, reducing both structural complexity and energy consumption while simultaneously improving aerodynamic performance [78,79,81].

Morphing aircraft can be classified according to the type of geometric transformation employed. Planform morphing modifies span, chord, or sweep to influence lift, induced drag, and maneuverability, while out-of-plane morphing involves twist, bending, or dihedral changes to improve load distribution and aeroelastic behavior. Airfoil morphing modifies camber or thickness to continuously control pressure distribution without the aerodynamic penalties associated with conventional flaps and slats. These deformation modes occur in both biological and engineered morphing wings, with their classification as passive or active depending on the mechanism generating the deformation. Span and variable-sweep morphing additionally enable adaptation of aspect ratio and wing geometry to balance performance across low- and high-speed flight conditions [78,79,80,82,83,84].

The successful implementation of these concepts requires structural solutions capable of simultaneously providing high load-carrying capacity and controlled flexibility. Consequently, modern morphing wings increasingly rely on compliant structures, anisotropic composite laminates, distributed stiffness tailoring, and flexible skins that allow deformation to be distributed throughout the wing instead of being concentrated at discrete hinges. These approaches closely parallel the hierarchical structural organization of insect wings, where vein architecture and heterogeneous material distribution generate controlled passive deformation under aerodynamic loading [79,80,81].

One of the most important engineering strategies inspired by biological passive morphing is aeroelastic tailoring, in which the structural response of the wing is programmed through the orientation and distribution of composite materials. By tailoring fibre orientations, laminate stacking sequences, or local stiffness distributions, engineers can promote beneficial bend–twist coupling, passive load alleviation, and adaptive deformation without continuous actuator input. Such designs exploit aerodynamic forces to generate favorable structural responses, representing one of the closest engineering analogues to the passive morphing mechanisms found in insect wings [81,83].

Recent developments have extended these concepts from experimental demonstrators toward practical aerospace applications. Adaptive wingtips, compliant trailing-edge devices, variable-camber airfoils, and passive load-alleviation systems have demonstrated measurable improvements in aerodynamic efficiency, fuel consumption, and structural loading. Nevertheless, current morphing aircraft remain significantly simpler than their biological counterparts. While insect wings integrate multiple adaptive functions through highly hierarchical vein networks and spatially varying material properties, most engineering solutions reproduce only one or two deformation mechanisms, highlighting the considerable opportunities that biological systems continue to offer for the development of future aerospace wing structures [78,79,80,81,82,83].

Passive morphing strategies have also stimulated the development of adaptive wing skins capable of accommodating large structural deformations while preserving a smooth aerodynamic surface. Unlike conventional metallic skins, which resist in-plane deformation, morphing skins must simultaneously exhibit high out-of-plane stiffness to withstand aerodynamic loading and low in-plane stiffness to permit controlled shape adaptation. To satisfy these conflicting requirements, researchers have proposed compliant honeycomb cores, elastomeric composites, anisotropic laminates, and cellular structures with tailored Poisson’s ratios. These architectures distribute deformation across the wing surface rather than concentrating it at discrete joints, allowing continuous changes in wing geometry that more closely resemble the membrane-supported deformation observed in insect wings [79,84].

Another important direction has been the development of variable-stiffness composite structures capable of reproducing the anisotropic mechanical behaviour characteristic of biological wings. Rather than relying on uniform laminate configurations, modern composite manufacturing techniques enable local tailoring of fibre orientation, fibre spacing, and stacking sequence to achieve spatially varying stiffness distributions. This approach allows engineers to program passive bend–twist coupling, control local deformation patterns, and improve aeroelastic performance under different loading conditions. Similar to insect wing venation, where stiffness gradually varies between veins and membranes, variable-stiffness laminates exploit structural anisotropy to guide deformation toward aerodynamically favourable configurations while maintaining sufficient load-carrying capacity [81,85].

The interaction between structural flexibility and aerodynamic loading has further encouraged the development of passive load-alleviation concepts in which the wing adapts automatically to changing flight conditions without continuous control inputs. Instead of resisting aerodynamic loads, these structures exploit them to generate beneficial deformation, reducing gust loads, delaying structural fatigue, and improving overall aerodynamic efficiency. Such aeroelastic behaviour represents one of the closest engineering analogues to passive morphing in insect wings, where aerodynamic forces continuously interact with heterogeneous structural properties to produce adaptive changes in twist, bending, and camber throughout the flapping cycle. Although current aerospace implementations remain considerably simpler than biological systems, recent advances in compliant structures, adaptive skins, and variable-stiffness composites demonstrate that many of the underlying mechanical principles governing passive morphing can be successfully translated into practical wing designs [81,82,83,84,85,86].

### 4.2. Bio-Inspired UAVs and MAVs

The rapid development of micro air vehicles (MAVs) and unmanned aerial vehicles (UAVs) has significantly increased interest in biological flight as a source of engineering inspiration. Operating at Reynolds numbers comparable to those experienced by flying insects, MAVs are strongly influenced by unsteady aerodynamic phenomena that cannot be efficiently exploited using conventional rigid-wing designs. Consequently, insect wings have become important biological models, not only because of their aerodynamic efficiency but also because of their ability to achieve passive shape adaptation through distributed flexibility and structural anisotropy. Rather than replicating biological morphology directly, current research increasingly seeks to translate the mechanical principles governing passive wing morphing into practical aerospace structures capable of improving flight efficiency, maneuverability, and robustness. Recent reviews further emphasize that the integration of biological principles with advanced computational design and modern manufacturing technologies has become one of the principal research directions in next-generation aerial robotics [87,88,89,90].

One of the most important concepts transferred from insect flight is the use of passive structural deformation to enhance aerodynamic performance. Unlike conventional aircraft, where wing deformation is generally minimized, insect-inspired MAVs intentionally exploit controlled flexibility to modify force generation throughout the flapping cycle. Passive bending, twisting, and camber variation modify the effective angle of attack and can influence flow separation, leading-edge vortex formation and evolution, and aerodynamic force generation without requiring additional actuation; however, these effects depend on wing geometry, structural stiffness, Reynolds number, and flapping kinematics. Numerical investigations of morphing wings have further demonstrated that passive structural deformation can influence aerodynamic performance through the coupling between structural response and unsteady flow evolution throughout the flapping cycle [87,88,91,92]. The translation of these biological principles into engineering structures has primarily focused on reproducing the anisotropic stiffness distribution characteristic of insect wings. Natural wings combine stiff longitudinal veins with highly compliant membranes, producing high spanwise rigidity while maintaining chordwise flexibility. This structural organization has inspired the development of rigid–flexible coupled wing architectures composed of lightweight composite spars, carbon-fibre reinforcements, polymer membranes, and compliant joints. Butterfly-inspired MAV studies have demonstrated that tailoring spanwise and chordwise stiffness distributions can influence thrust generation, flight stability, and aerodynamic efficiency, while recent optimization studies indicate that wing curvature and flexibility can be systematically adjusted to improve aerodynamic performance under specific operating conditions. Fluid–structure interaction analyses further show that optimal stiffness distributions closely resemble those measured in natural butterfly wings, highlighting the importance of structural anisotropy for passive morphing performance [88,93,94].

Advances in computational design have accelerated the development of bio-inspired aerial vehicles by integrating biological observations with numerical optimization. Parametric wing models, finite element analyses, computational fluid dynamics, and fluid–structure interaction simulations are increasingly combined to determine optimal membrane thickness, vein arrangement, and stiffness distributions before prototype fabrication. Rather than directly copying insect wings, these methods identify the biomechanical principles responsible for favourable passive deformation and translate them into manufacturable engineering designs. Numerical models of bio-inspired morphing wings have demonstrated good agreement with experimental observations, confirming that coupled structural and aerodynamic simulations provide reliable tools for optimizing passive morphing configurations before experimental validation [92,94].

Recent fabrication technologies have also enabled increasingly realistic reproduction of insect wing architectures. Microfabrication techniques, laser micromachining, additive manufacturing, flexible polymers, and composite materials now allow the fabrication of lightweight artificial wings with embedded compliant elements and vein-like supporting structures. These manufacturing approaches provide greater control over membrane thickness, vein geometry, and stiffness distribution, allowing engineers to systematically investigate the influence of structural parameters on passive morphing behaviour. Dragonfly-inspired additively manufactured wings have demonstrated favourable combinations of lightweight construction, structural resilience, and aerodynamic efficiency, while folding-wing concepts inspired by insect wing deployment have introduced compact mechanisms capable of improving portability and mission adaptability in small aerial vehicles [95].

Beyond improving aerodynamic efficiency, passive morphing also contributes to flight robustness. Biological flyers continuously adapt to gusts and external disturbances through their intrinsic structural compliance, reducing the need for rapid active control. This concept has inspired soft aerial robots and compliant-wing MAVs capable of absorbing transient aerodynamic loads while maintaining stable flight. Adaptive morphologies reduce control effort, improve disturbance rejection, and enhance operational reliability, particularly in cluttered or turbulent environments where conventional rigid-wing vehicles experience significant performance degradation. Bird-inspired morphing mechanisms have additionally demonstrated that combining passive structural adaptation with limited active control can substantially expand the flight envelope of small UAVs, illustrating how biological concepts from both insect and avian flight continue to influence modern morphing-wing design [96,97].

Despite the substantial progress achieved over the past two decades, current bio-inspired UAVs and MAVs still reproduce only a limited subset of the multifunctionality observed in insect wings. Natural wings integrate passive deformation, heterogeneous material properties, hierarchical venation networks, sensing capabilities, and aerodynamic optimization within a single lightweight structure. Engineering implementations generally simplify these features to improve manufacturability and structural reliability. Nevertheless, recent developments in computational optimization, multifunctional materials, soft robotics, additive manufacturing, and high-fidelity aeroelastic modelling are progressively narrowing this gap, moving bio-inspired aerial vehicles beyond simple geometric imitation toward functional biomimicry, where the passive morphing mechanisms responsible for the exceptional performance of insect wings are systematically incorporated into aerospace structures.

### 4.3. Smart Materials and Adaptive Structures

Translating the multifunctional and self-adaptive characteristics of natural flyers into engineered aerospace systems increasingly relies on compliant materials, architected structures, and distributed stiffness rather than exclusively on rigid components and discrete mechanical linkages [12,98,99,100,101,102,103]. In the context of the classification introduced above, these technologies can contribute either to passive morphing, where deformation results primarily from aerodynamic or inertial loading, or to hybrid morphing, where limited active input modifies or regulates an intrinsically compliant structural response. Smart materials are therefore most relevant to the present review when they support structural compliance, stiffness tailoring, multistability, sensing, or limited actuation within such passive or hybrid architectures [12,95,100].

Active smart materials remain useful primarily as enabling components of hybrid morphing systems. Shape Memory Alloys (SMAs), for example, combine relatively large recoverable strains with high actuation energy density and have been investigated for variable-camber, twist, and other slowly varying morphing configurations [13,100]. Their thermally driven response, however, limits actuation bandwidth and makes them more appropriate for configuration changes or structural-state adjustment than for reproducing the rapid passive deformation characteristic of insect wings [12,101,102]. Piezoelectric materials provide a complementary capability because of their rapid electromechanical response, although monolithic ceramics generally offer limited strain and can be mechanically brittle [103,104,105]. Macro-Fiber Composites overcome some of these limitations by embedding piezoceramic fibers within a flexible polymer matrix, allowing distributed bending and twisting when incorporated into compliant structures [99,100,101]. In a hybrid morphing system, such actuators can be used to trim the wing, modify its effective stiffness, or initiate a structural transition rather than prescribing the complete deformation continuously.

Passive morphing is more directly associated with structural architecture and spatial stiffness tailoring. Cellular and composite structures can be designed to remain sufficiently stiff against aerodynamic pressure while allowing large deformation along selected directions [99,100]. Auxetic architectures, for example, exhibit a negative Poisson’s ratio and can accommodate substantial in-plane deformation [96], whereas near-zero-Poisson-ratio cellular structures can reduce unwanted transverse distortion during spanwise or chordwise morphing [100,101,102,103,104]. These concepts provide a functional engineering analogue to insect-wing venation, where heterogeneous stiffness and structural geometry guide deformation under external loading rather than relying on distributed active actuation.

Multistable and bistable structures provide another bridge between passive and hybrid morphing. Prestressed or thermally induced composite laminates can possess two or more stable equilibrium configurations, allowing the structure to transition rapidly between predefined shapes [100]. When the transition is triggered directly by aerodynamic loading, the concept can provide passive adaptation or load alleviation; when a small SMA or piezoelectric input is used to initiate the transition, it becomes a hybrid system [100,101]. The principal advantage is that continuous actuator power is not necessarily required to maintain each stable configuration, reducing the actuation demand relative to continuously driven morphing.

Manufacturing technologies further enable these structural concepts. Origami- and kirigami-inspired architectures, compliant flexures, and multi-material additive manufacturing can integrate rigid load-bearing regions with flexible hinges or compliant zones, producing lightweight structures with controlled folding and deformation [10,105]. Flexible morphing skins remain particularly challenging because they must accommodate substantial in-plane strain or camber variation while simultaneously resisting aerodynamic pressure, local buckling, and surface distortion [99,106]. Cellular substrates, elastomeric skins, and variable-stiffness composite concepts have therefore been investigated to reconcile these competing requirements [99,104]. Their relevance to insect-inspired morphing lies in distributing stiffness and compliance throughout the structure so that deformation occurs preferentially where it provides an aerodynamic benefit.

Embedded sensing can further support hybrid adaptive structures by providing information about the deformation and aerodynamic state without replacing the underlying passive mechanical response. Inspired in part by distributed biological sensing systems [10,102], engineered morphing structures increasingly incorporate strain and shape measurements for structural monitoring and feedback control [19,100,107]. Fiber Bragg Grating sensors and related distributed sensing technologies can provide information on wing deformation and structural loading during operation [108]. Such feedback can be used to apply only the active correction required to regulate an otherwise compliant structure, thereby combining the low-energy response of passive morphing with the controllability of active systems.

Overall, smart materials are most relevant to insect-inspired morphing when they are treated not as substitutes for passive aeroelasticity, but as tools for creating distributed compliance, spatial stiffness tailoring, multistability, sensing, and limited active regulation. Passive systems derive their deformation primarily from aerodynamic and inertial loads, whereas hybrid systems use smart materials or actuators to adjust, initiate, or stabilize this load-driven response. This distinction maintains the connection to insect wings, where adaptive deformation emerges predominantly from structural geometry, heterogeneous material properties, and fluid–structure interaction.

### 4.4. Engineering Challenges

Despite substantial advances in bio-inspired morphing structures, translating the passive adaptability of insect wings into operational aerospace systems remains a major multidisciplinary challenge. Biological wings achieve deformation, load transfer, damage tolerance, and environmental resilience through a highly integrated architecture of veins, membranes, joints, and heterogeneous materials. Engineered morphing wings, in contrast, must reproduce selected aspects of this functionality while satisfying stringent requirements related to structural strength, aerodynamic accuracy, manufacturability, controllability, durability, and certification. The principal limitations can be grouped into five closely connected areas: scaling laws, manufacturing, materials, control, and reliability [109,110,111,112,113].

#### 4.4.1. Scaling Laws

One of the most fundamental difficulties in transferring insect-wing mechanisms to aerospace structures is the absence of direct geometric and dynamic scalability. Insect flight generally occurs at low Reynolds numbers, where viscous effects, unsteady separation, leading-edge vortices, wing rotation, wake capture, and fluid–structure interaction dominate aerodynamic force production. When a biological wing architecture is enlarged or transferred to a fixed-wing vehicle, the relative contributions of aerodynamic, inertial, elastic, and gravitational forces change substantially. Consequently, a structure that generates beneficial passive twist or camber at insect scale may become excessively compliant, insufficiently responsive, or structurally impractical at larger dimensions [109,111].

Geometric similarity alone is therefore insufficient to guarantee dynamically similar aerodynamic behavior. Experimental studies of geometrically similar three-dimensional flapping wings have shown that force coefficients remain sensitive to Reynolds number, reduced frequency, the location of the flapping center, minimum wing-to-wing separation, and the choice of reference velocity used for normalization. In particular, the velocity evaluated at the radius of the second moment of wing area provides a more consistent basis for force scaling than the average wingtip velocity when comparing different wing sizes and rotational configurations [111]. Differences in wake capture, wing rotation, wing–wing interaction, and leading-edge vortex development may still produce dissimilar force histories even when the wing planform and prescribed kinematics are geometrically scaled.

Scaling also affects the balance between aerodynamic loading and structural deformation. Low-Reynolds-number studies indicate that flexibility can smooth lift fluctuations, promote passive twist, and alleviate gust loads, but increasing compliance does not necessarily improve all performance measures simultaneously. Depending on the structural stiffness and flapping kinematics, greater flexibility may increase thrust while reducing lift or may generate excessive phase lag between deformation and aerodynamic loading [109]. The response is therefore governed by nondimensional aeroelastic parameters rather than geometry alone.

The same difficulty arises when experimental results from wind-tunnel demonstrators are extrapolated to full-scale aircraft. Passive morphing airfoils tested at Reynolds numbers of approximately 10^5^ have demonstrated load-induced camber changes and increased lift slopes, but their behavior depends strongly on viscous effects. RANS-based aeroelastic simulations reproduce measured deformation more accurately than inviscid Euler solutions, which tend to overestimate aerodynamic loading and structural displacement [112]. Similarly, passive gust-alleviation experiments on high-aspect-ratio wings cannot be directly transferred to full-scale high-altitude aircraft because changes in Reynolds number, material nonlinearity, structural damping, and mass ratio may alter the flutter boundary and the triggering conditions of multistable components [113]. Reliable bio-inspired scaling must therefore preserve the governing aerodynamic and structural similarity parameters, not merely the external geometry.

#### 4.4.2. Manufacturing

The hierarchical organization of insect wings is difficult to reproduce using conventional aerospace manufacturing. Biological wings contain curved and tapered veins, local thickness variations, compliant joints, corrugated membranes, and gradual stiffness transitions over length scales that differ by several orders of magnitude. Conventional machining and composite lay-up processes are generally optimized for relatively uniform components and may require numerous parts, joints, molds, and assembly operations to approximate such architectures. These additions increase mass, introduce backlash and stress concentrations, and reduce the mechanical simplicity that originally motivates passive morphing [114,115].

Additive manufacturing provides a promising route to complex compliant mechanisms, lattice structures, graded architectures, and integrated morphing components. Continuous-fibre composite printing has already been used to manufacture the primary and morphing structures of a two-metre-span fixed-wing drone that was subsequently flight-tested. Local fibre placement enabled the structural anisotropy and load paths to be tailored while reducing part count and eliminating conventional hinged control surfaces [108]. Fully printed compliant mechanisms have also demonstrated substantial trailing-edge deflections and rapid design iteration using low-cost polymer extrusion [114,115]. However, the mechanical properties of printed components are highly dependent on build orientation, layer adhesion, void content, fibre continuity, processing temperature, and local geometric resolution. These manufacturing effects must be represented in structural and aeroelastic models if simulation results are to predict the behaviour of the fabricated wing accurately [114,116].

The manufacture of variable-stiffness composites presents additional constraints. Curvilinear fibre paths and variable-angle tow laminates can guide deformation and provide beneficial bend–twist coupling, but tow steering may introduce fibre waviness, gaps, overlaps, variations in fibre volume fraction, and residual stresses. Although fibre continuity can increase the load-carrying capability of variable-angle laminates, the manufactured stiffness distribution cannot always reproduce the mathematically optimal distribution exactly [117,118]. Optimized designs must therefore incorporate minimum turning radii, allowable tow spacing, ply-drop rules, laminate blending, and other process constraints from the beginning of the design cycle.

Morphing skins remain particularly difficult to manufacture. A skin must maintain a smooth aerodynamic surface and carry pressure loads while permitting large in-plane deformation with limited actuation force. Sliding composite skins can reduce wrinkling and sagging without requiring large material strains, but they introduce guides, contact interfaces, friction, and sealing problems [119]. An alternative approach is the use of multi-material skins that combine flexible elastomeric regions with stiffer fibre-reinforced structures. Falken et al. [120] investigated such a concept for morphing aircraft, highlighting the challenges associated with load introduction, manufacturing, cyclic loading, and the trade-off between structural strength and morphing capability. Their work demonstrates that, although multi-material skins can provide the flexibility required for large deformations, their practical implementation requires careful material selection, structural integration, and manufacturing control.

The principal advantages, limitations, and potential roles of these material systems in morphing-wing structures are compared in Table 7. The comparison confirms that no individual material satisfies all structural, deformation, durability, and operational requirements, supporting the development of hybrid systems with spatially distributed functions.

#### 4.4.3. Control

Passive morphing reduces the need for continuous actuation, but it does not eliminate the control problem. Because deformation is generated through coupling between external loads and structural compliance, the wing response cannot be commanded independently of airspeed, angle of attack, gust intensity, structural temperature, or dynamic pressure. A stiffness distribution optimized for one condition may produce insufficient adaptation at low loads and excessive deformation or instability at higher loads. Passive systems must therefore be designed to exhibit a predictable and beneficial response throughout the operating envelope [112,113].

Hybrid systems combining passive load response with sensing and limited active correction may offer a more practical solution. Distributed strain and piezoelectric sensor networks have demonstrated the ability to estimate airspeed, angle of attack, stall onset, and morphing-wing shape in near real time. Machine-learning models applied to a variable-camber wing achieved a stall-classification validation accuracy of approximately 99.4% and operated at 30 Hz, although reliability of the smart-material actuator limited the available morphing range during dynamic testing [127,128,129,130,131]. Such fly-by-feel architectures imitate the distributed sensing of biological wings by using structural deformation and vibration as sources of flight-state information.

The integration of deep learning with morphing wings may also reduce sensing and computational requirements. Wind-tunnel experiments using end-to-end deep reinforcement learning achieved approximately 84–86% gust rejection using pressure measurements from a camber-morphing wing. Controllers supplied by only three pressure taps performed statistically similarly to those using six taps, indicating that carefully selected sparse sensing can retain most of the information required for control [121]. Non-uniform spanwise camber control has additionally identified configurations that reduce drag during specific gust conditions, although its advantage over uniform morphing was not universal and was demonstrated over a restricted operating condition [129].

High-fidelity CFD–FSI models are generally too computationally expensive for onboard control. Reduced-order and data-driven models are therefore necessary to predict nonlinear aeroelastic behaviour in real time. Parametric models based on Dynamic Mode Decomposition with control have reproduced nonlinear lift and drag responses with errors below approximately 2% over multiple operating regimes while operating more than three orders of magnitude faster than the full-order model. When integrated with model-predictive control, such models were able to mitigate gust-induced load peaks that could not be controlled using a single non-parametric model [128]. Nevertheless, data-driven controllers remain sensitive to training-data coverage, sensor noise, material aging, and configurations outside the calibrated range. Their use in safety-critical flight will require uncertainty quantification, physical constraints, explainable failure behaviour, and reliable fallback control laws.

Reliability remains a major barrier between morphing-wing demonstrators and operational aircraft, as repeated deformation, aerodynamic loading, vibration, environmental exposure, and impact can progressively degrade skins, joints, sensors, actuators, and interfaces [120,122]. Insect wings offer relevant durability principles through their hierarchical venation, compliant joints, surface properties, and crack-arresting mechanisms, suggesting that durability should be considered at the system level rather than for individual components alone [129]. Morphing structures also introduce additional aeroelastic risks, as actuator stiffness and failure, free-play, mass distribution, and interface properties can influence flutter behaviour, while designs optimized for static conditions may violate structural constraints under dynamic gust loading [118,130].

Operational implementation therefore requires predictable relationships between aerodynamic loading, deformation, structural integrity, and control response throughout the flight envelope. Long-duration testing, structural health monitoring, fail-safe mechanisms, and consideration of manufacturing and environmental uncertainties will be essential for certification. Ultimately, successful bio-inspired morphing requires translating not only the adaptability of insect wings but also their distributed robustness into structures that remain predictable, inspectable, repairable, and certifiable throughout their operational life [129,130,131].

## 5. Future Perspectives and Conclusions

### 5.1. Research Gaps

Despite substantial progress in understanding biological wing deformation and developing adaptive aerospace structures, several fundamental gaps continue to limit the systematic translation of passive morphing principles into practical engineering applications. These gaps concern not only the maturity of individual technologies but also the comparability of experimental results, the characterization of materials under realistic operating conditions, and the integration of biological, structural, aerodynamic, and control models within unified predictive frameworks.

A major limitation in passive-morphing research is the absence of standardized, openly accessible datasets integrating wing morphology, material properties, deformation, aerodynamic loading, and flow-field measurements. Differences in species, geometry, boundary conditions, experimental procedures, kinematics, and measurement techniques make direct comparison and reproducibility difficult [45,46,54,56,57]. Even geometrically similar wings may exhibit different aerodynamic responses when Reynolds number, reduced frequency, flapping-center position, wing interactions, and reference velocities are not consistently defined [111]. Consequently, distinguishing genuine physical mechanisms from biological or methodological variability remains challenging.

Standardized benchmark datasets should therefore combine three-dimensional wing geometry and material properties with synchronized measurements of deformation and aerodynamic forces [55,56,57], together with quantitative characterization of the surrounding flow field [59,60,61,62]. Complete reporting of boundary conditions, calibration procedures, and measurement uncertainty would further improve reproducibility and facilitate comparison among experimental configurations. Such benchmarks would enable more consistent validation and comparison of FEM, CFD, and coupled FSI models [69,70,71]. They are particularly important for data-driven approaches, for which predictive performance depends strongly on the diversity and representativeness of the training data [124,126,128]. Datasets spanning multiple geometries and operating conditions are therefore needed to assess model generalization, uncertainty, and transferability.

Material characterization remains another major limitation in passive-morphing research. Insect wings are heterogeneous structures combining sclerotized cuticle, resilin-rich joints, tubular veins, membranes, corrugations, and spatial variations in thickness and stiffness [24,25,26,27,28,29,30,31]. Experimental investigations have further demonstrated substantial spatial variation in wing structural and mechanical properties [58,59,60,61,62,63]. Nevertheless, available material data cover relatively few species and wing regions, leading many computational models to adopt homogeneous, isotropic, or linearly elastic approximations despite the anisotropic, nonlinear, and heterogeneous behaviour of biological wings. Biological variability, specimen preparation, hydration, and testing conditions further complicate comparison, while properties such as damping, fatigue resistance, fracture toughness, and damage evolution remain insufficiently characterized [132].

Similar limitations affect engineered morphing materials. Elastomeric skins and variable-stiffness or multistable composites exhibit performance that depends on material properties, loading conditions, hysteresis, and fatigue [114,115,116,133], while additively manufactured and smart morphing structures introduce additional sensitivity to manufacturing parameters, material interfaces, and imperfections [122,123,124,125]. Component-level performance alone is therefore insufficient to predict the behaviour and durability of an integrated morphing structure [131]. Future research should focus on standardized multiscale characterization, from constituent materials and interfaces to complete morphing assemblies, incorporating mechanical, environmental, and fatigue testing together with uncertainty distributions for reliable prediction of deformation, damage, and service life.

Another major research gap is the absence of a unified modelling framework connecting morphology, material heterogeneity, structural deformation, aerodynamic loading, and control. Existing approaches address only parts of this coupled problem: analytical and reduced-order models require substantial simplification [66], FEM provides greater structural detail [67,68], and CFD/FSI captures complex aerodynamic and aeroelastic interactions at considerably higher computational cost [69,70,71,72]. Moreover, material nonlinearities, damping, joint compliance, manufacturing imperfections, and transient morphing effects are frequently simplified, despite evidence that aerodynamic-model fidelity and actuation dynamics can significantly influence predicted deformation and aerodynamic performance [109,112,113].

Future frameworks should therefore integrate structural and aerodynamic models with experimental data, manufacturing constraints, sensing, optimization, and uncertainty quantification. Existing optimization approaches already incorporate variable stiffness, manufacturability, gust loading, and topology optimization [118,121], while data-driven reduced-order models and machine-learning methods offer substantial gains in computational efficiency and real-time state estimation [124,126,128]. Combining these approaches through physics-informed or hybrid modelling could enable efficient prediction of nonlinear aeroelastic behaviour, stability, and transient deformation, allowing passive morphing to be designed as an integrated and predictable system-level capability.

### 5.2. Emerging Research Directions

The future development of passive morphing systems will depend on the convergence of computational intelligence, advanced manufacturing, multifunctional materials, embedded sensing, and application-specific design. Rather than reproducing insect-wing morphology directly, emerging research increasingly seeks to transfer the underlying principles of distributed compliance, structural anisotropy, passive load adaptation, hierarchical organization, and multifunctionality into engineered systems. This transition is expected to produce morphing structures that are not only aerodynamically efficient, but also manufacturable, observable, damage-tolerant, and capable of adapting across multiple operating environments.

Artificial intelligence offers a promising route for reducing the computational cost of coupled aerodynamic and structural analyses of morphing wings. Data-driven surrogate and reduced-order models can approximate relationships among geometry, deformation, flow conditions, and aerodynamic response, enabling faster design exploration and optimization. For example, machine-learning-assisted reduced-order modelling has reproduced turbulent flow around dynamically morphing aerofoils with errors below 3%, demonstrating the potential to complement computationally expensive CFD and FSI simulations [134].

**Figure 6:**
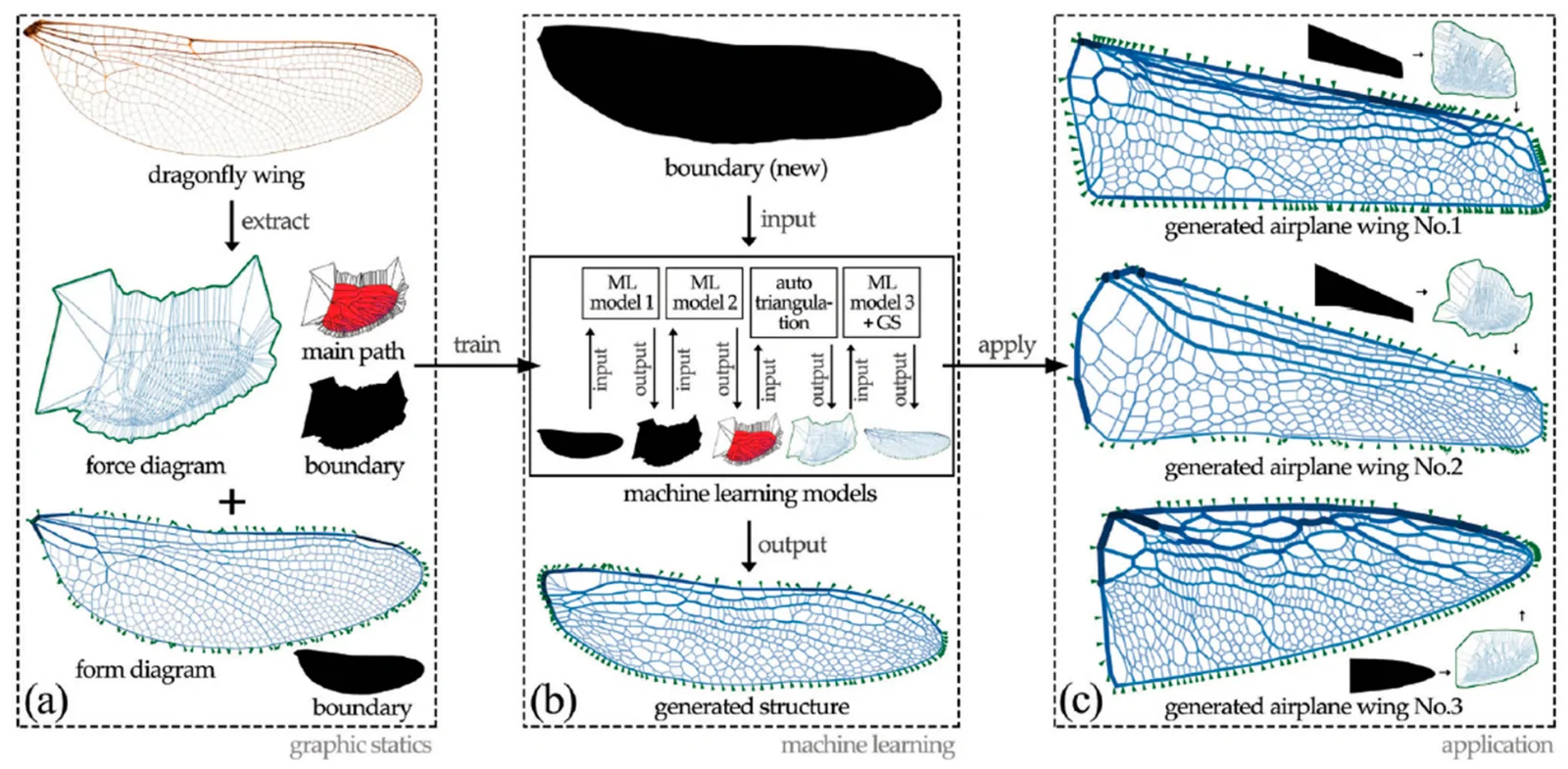
Workflow of AI-assisted biomimetic design inspired by dragonfly wing venation: (**a**) extract the main features of the dragonfly wings using graphic statics; (**b**) train machine learning models using the form and force diagrams to generate dragonfly wing structures; (**c**) implement the trained models from step (**a**) to generate a determinate network for given boundary conditions of an airplane wing structures [135].

AI can also facilitate the translation of biological principles into engineering designs. Zheng et al. [135] combined machine learning with Maxwell’s reciprocal diagrams to generate lightweight cellular structures inspired by dragonfly-wing venation (Figure 6), achieving improvements of up to 25% in out-of-plane stiffness compared with conventional designs. Rather than directly reproducing biological geometry, such approaches extract and optimize its underlying structural principles. AI-assisted modelling could therefore accelerate the exploration of stiffness distributions, venation-inspired architectures, and morphing configurations while reserving high-fidelity simulations and experiments for final validation.

**Figure 7:**
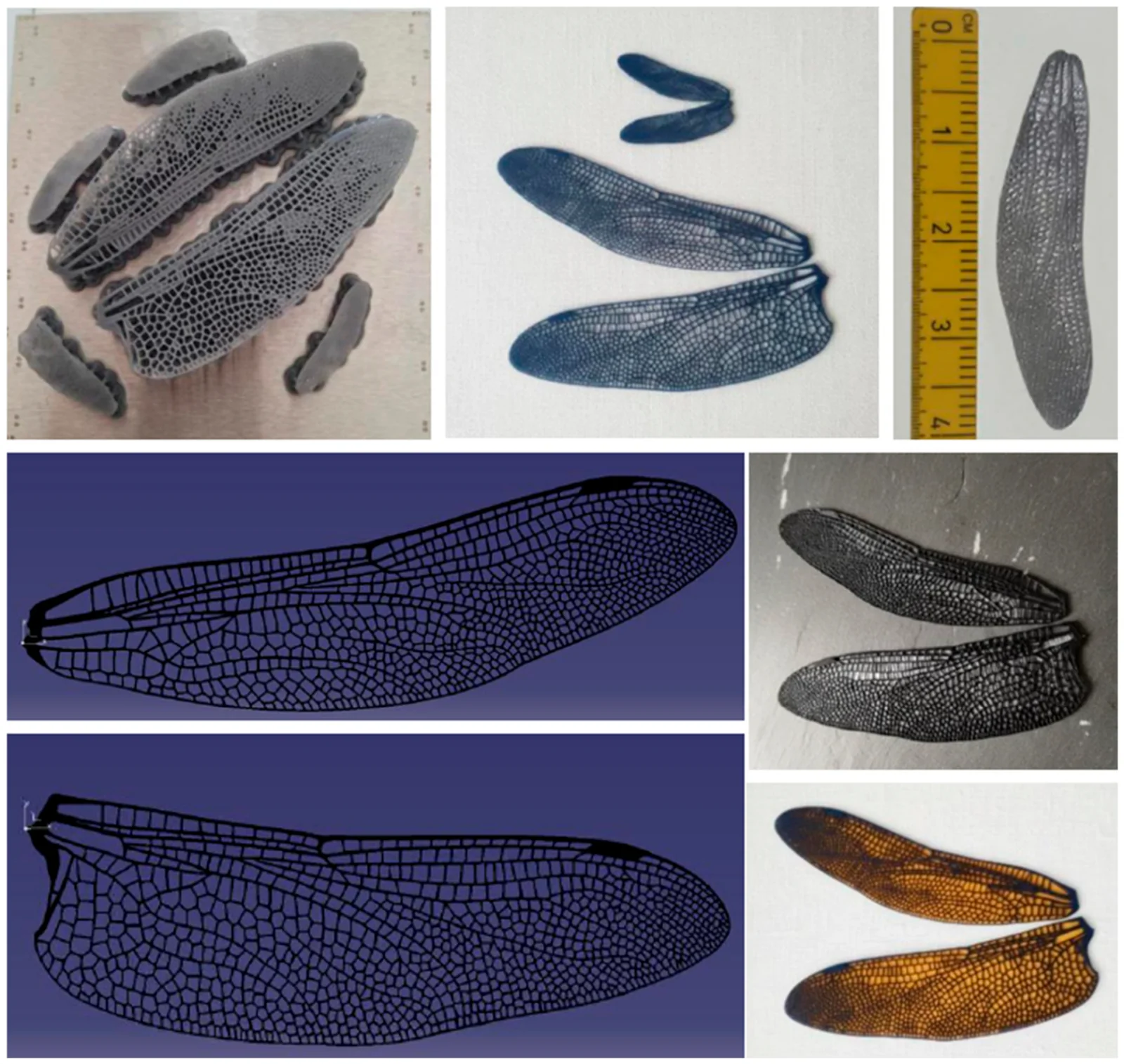
From CAD modelling to 3D manufacturing of dragonfly wings [93].

Additive manufacturing provides an effective route for reproducing the geometric complexity and distributed stiffness of insect wings by integrating compliant regions, stiff load paths, lattice networks, and spatially varied material properties within a single structure. This is particularly valuable for passive morphing, where deformation can be programmed through geometry and material distribution. High-resolution biological geometries can also be digitally reconstructed and directly fabricated, preserving hierarchical venation and anisotropic structural behaviour. For example, additively manufactured dragonfly-wing models at biological and enlarged scales have been developed as experimental platforms for modal, aeroelastic, and flow investigations (Figure 7) [95].

Beyond biological replication, additive manufacturing enables rapid design–manufacture–test cycles in which venation patterns, membrane thicknesses, and structural configurations can be systematically varied and experimentally evaluated. This flexibility provides a practical pathway from biological observation to lightweight adaptive structures and bio-inspired micro air vehicles.

**Figure 8:**
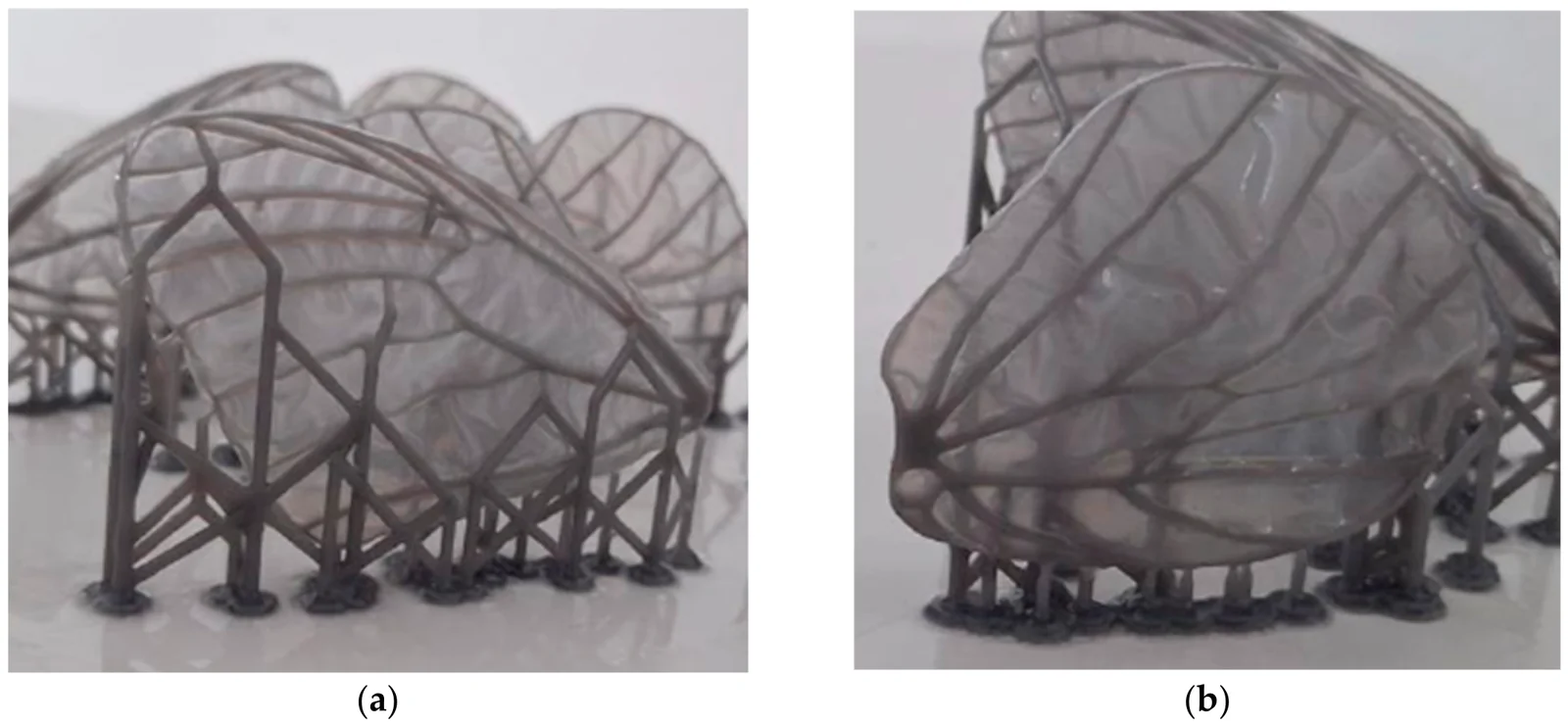
Polymeric resin manufactured (**a**) forewing and (**b**) hindwing of the *Idea leuconoe* [56].

An example of this approach is illustrated in Figure 8, where butterfly forewing and hindwing models inspired by *Idea leuconoe* were reconstructed in CAD and fabricated using stereolithography [56]. The models preserve the principal venation architecture while providing a simplified platform for finite element analysis, modal characterization, and future aerodynamic testing. The use of additive manufacturing enabled rapid optimization of the prototype geometry, including multiple printing iterations and subsequent structural evaluation, demonstrating how biological observations can be efficiently transformed into experimentally validated engineering concepts for flapping-wing micro air vehicles.

Zhao et al. [132] demonstrated the potential of machine learning for aerodynamic prediction and autonomous morphing control. The DLCN model incorporates physical monotonicity constraints to improve the accuracy, reliability, and interpretability of lift and drag predictions, while the DDPGwTC framework combines DDPG and LSTM networks to enable efficient, adaptive selection of optimal morphing configurations across different mission environments [136].

Machine learning-assisted design and additive manufacturing have enabled dragonfly-inspired wings with metamaterial reinforcement patterns, demonstrating that biologically inspired anisotropy can be achieved through controlled geometry and local cellular architecture rather than multiple materials [137]. Four-dimensional printing extends this concept by enabling structures to transform after fabrication; adaptive trailing-edge prototypes have combined load-bearing capability with flexion angles of up to 20° [138]. Similarly, multi-material 3D-printed variable-camber wings have achieved substantial trailing-edge deformation while maintaining continuous aerodynamic surfaces, while gradient metamaterials and continuous-fibre additive manufacturing offer further opportunities for lightweight adaptive structures [138,139].

Future research should focus on repeatable production of functionally graded morphing structures, particularly regarding interface reliability, fibre-path control, dimensional accuracy, embedded sensing, repairability, and deformation repeatability. Process-aware models will also be needed to incorporate manufacturing variability directly into structural and aerodynamic predictions.

Digital twins, multifunctional structures, and planetary applications represent complementary future directions for bio-inspired morphing. Digital twins could connect experimental observations with predictive models by continuously updating structural and aerodynamic parameters using distributed measurements of pressure, strain, deformation, temperature, vibration, and damage. Emerging frameworks combining adaptive digital twins, reinforcement learning, and flexible sensing demonstrate the potential of this approach for flight control and structural monitoring [140,141]. Coupled with reduced-order models and periodic high-fidelity simulations, such systems could support morphing-wing optimization and health assessment throughout their operational lifecycle.

At the structural level, insect wings demonstrate how load bearing, aerodynamic shaping, sensing, damping, and damage resistance can be integrated within lightweight architectures. Biomimetic materials increasingly combine self-sensing, self-healing, shape-memory, anti-icing, and damage-resistant functions [142], while experimental bio-inspired wings have integrated aerodynamic performance with sensing, energy harvesting, and active shape adaptation [142,143]. Hybrid architectures combining predominantly passive deformation with low-power active materials may therefore provide a practical compromise between energy efficiency and controllability.

These capabilities may be particularly valuable for planetary flight, where severe constraints on mass, packaging volume, atmospheric density, temperature, and available power favour lightweight and adaptive structures. Deployable morphing concepts have been proposed for Mars and Titan exploration to combine compact storage with aerodynamic adaptation and multifunctional mobility [144,145]. Marsbee experiments further demonstrated the feasibility of insect-inspired flapping flight under simulated Martian atmospheric density, while highlighting uncertainties in passive deformation under low-density conditions [146]. However, planetary morphing systems remain at an early validation stage and require testing under representative atmospheric, thermal, radiation, dust, and reduced-gravity conditions [146]. Overall, passive and hybrid morphing could reduce actuator complexity and power demand while providing the adaptability required for uncertain extraterrestrial environments. The maturity and principal challenges of these emerging research directions are summarized in Table 8.

## 6. Conclusions

Passive morphing in insect wings demonstrates how substantial aerodynamic adaptability can emerge from the interaction of structural geometry, material heterogeneity, and external loading without continuous mechanical actuation. Wing deformation is not governed by flexibility alone but by the coordinated arrangement of veins, membranes, compliant joints, corrugations, and spatial stiffness gradients. Together, these features produce controlled bending, twisting, and camber variation while maintaining the structural integrity required for repeated flapping cycles [32,35,36]. The principal engineering lesson is therefore not the direct reproduction of insect-wing morphology, but the deliberate programming of structural response through geometry and distributed mechanical properties.

Experimental and computational research has significantly improved the understanding of these mechanisms. High-speed imaging, DIC, PIV, Schlieren visualization, microscopy, and force measurements provide complementary information on wing motion, deformation, aerodynamic loading, and flow development [44,45,46,47,48,49,50,51,52,53,54,55,56,57,58,59,60,61,62,63,64,65]. Analytical models, FEM, CFD, and FSI simulations have extended this understanding by linking structural architecture to aerodynamic performance [66,67,68,69,70,71,72,73,74,75,76]. Nevertheless, differences in biological specimens, testing conditions, material assumptions, and numerical formulations continue to limit comparisons among studies. Standardized datasets and validation procedures remain essential for distinguishing general passive-morphing principles from responses specific to an individual species, geometry, or experimental configuration.

The translation of these principles into aerospace structures has produced compliant trailing edges, flexible MAV wings, variable-stiffness composites, lattice structures, smart skins, and bio-inspired flapping-wing vehicles. These developments confirm that distributed compliance can improve aerodynamic adaptation while reducing geometric discontinuities and, in some cases, mechanical complexity. However, the benefits demonstrated by individual components do not yet guarantee the viability of an integrated aircraft. Manufacturing tolerances, fatigue, environmental degradation, sensing requirements, stability, maintainability, and certification must be considered together with aerodynamic efficiency. Passive deformation must remain beneficial and predictable throughout the complete flight envelope rather than only at a selected design condition [113,118,120].

**Figure 9:**
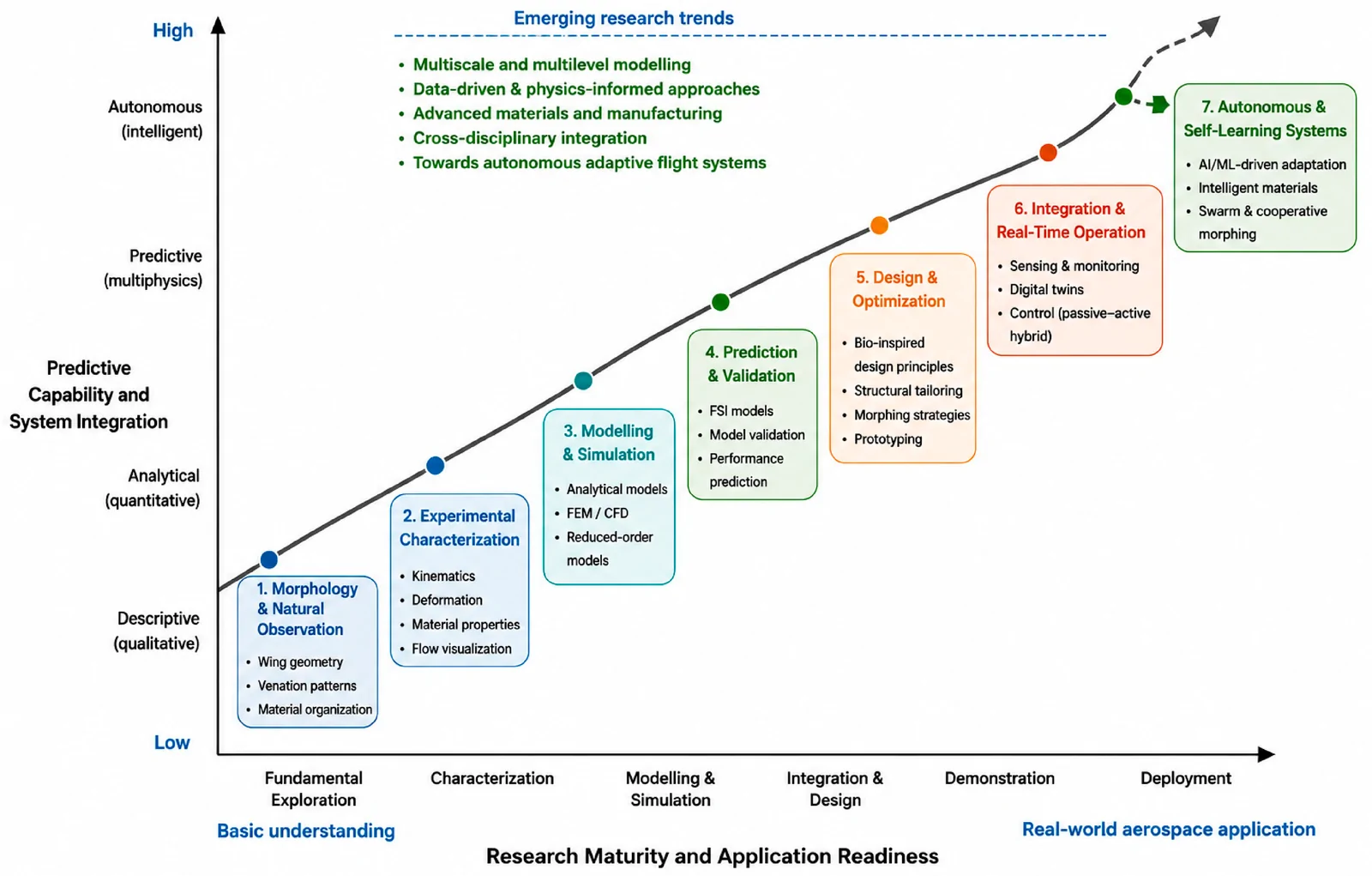
Conceptual evolution of passive morphing research with increasing predictive capability, system integration, and application readiness, from morphological observation to autonomous adaptive aerospace systems.

Figure 9 provides a conceptual representation of the evolution of passive morphing research from descriptive biological observation toward increasingly predictive and integrated aerospace applications. The progression reflects the growing use of quantitative experiments, computational modelling, fluid–structure interaction, optimization, digital twins, and intelligent adaptive systems. Rather than representing a strict chronological sequence, the graph illustrates increasing research maturity, predictive capability, and application readiness across the field.

Emerging technologies may help bridge the gap between biological understanding and engineering implementation. AI-assisted reduced-order models can accelerate aerodynamic and aeroelastic prediction, while additive and 4D manufacturing can enable structures with spatially tailored stiffness [131,136,137,138]. Digital twins and distributed flexible sensing could support model updating, damage detection, state estimation, and adaptive control [140,141], while multifunctional materials may integrate morphing, sensing, energy harvesting, and environmental protection within load-bearing structures [142,143]. However, the practical value of these technologies will depend on whether such integration reduces overall system mass, energy demand, and complexity without introducing additional failure modes.

Planetary flight particularly illustrates both the potential and the limitations of bio-inspired morphing. Deployable wings, soft morphing robots, and insect-inspired flapping vehicles could provide compact packaging and adaptive mobility in extraterrestrial environments [144,145,146]. However, passive deformation under ultra-low atmospheric density demonstrates that insect-scale aerodynamic behaviour cannot be assumed to persist under substantially different operating conditions [138]. The engineering relevance of insect wings therefore lies not in the direct scaling or replication of their aerodynamic behaviour, but in the transfer of underlying mechanical principles, such as distributed compliance, structural anisotropy, stiffness gradients, and load-dependent deformation. These principles must be re-optimized and validated for the aerodynamic, structural, material, and operational conditions of the target vehicle.

Overall, bio-inspired passive morphing should therefore be regarded as a design paradigm rather than a directly scalable biological solution. Its potential is particularly relevant to MAVs, UAVs, and other vehicles subject to strict mass and power constraints, but its broader aerospace applicability requires further validation across relevant scales and operating conditions. Future development will require closer integration of biological characterization, predictive modelling, experimental validation, advanced manufacturing, and lifecycle assessment. Hybrid architectures may provide a particularly practical pathway, allowing passive structural adaptation to provide much of the required deformation while limited sensing or actuation supplies control and shape retention. Such an approach could retain the low-energy and structurally efficient characteristics of passive morphing while providing the predictability and reliability required for aerospace operation.

## Figures and Tables

**Table 2:** Mechanical properties of the principal structural materials found in insect wings.

Material/Structure	Mechanical Characteristic	Main Function	Contribution to Passive Morphing	References
Resilin-rich regions	Highly compliant and elastically recoverable	Flexible joints	Local compliance and elastic recovery	[31,32,33]
Wing membrane	Thin, flexible cuticular structure	Aerodynamic surface and load transfer	Camber and local deformation	[30,34]
Sclerotized cuticle	Comparatively stiff cuticular material	Structural reinforcement	Maintains load-bearing capability	[30,33]
Veins	Geometry- and location-dependent stiffness	Load-bearing skeleton	Controls spanwise/chordwise stiffness and torsional response	[30,33,35,36]

**Table 3:** Mechanical Deformation Mechanisms in Insect Wings.

Mechanism	Functional Significance	Structural Basis	References
Twist	Modifies local wing orientation during the flapping cycle	Torsional compliance and spatial stiffness distribution	[30,35,36,37,38,39,40]
Camber	Produces adaptive sectional curvature	Chordwise compliance and vein–membrane architecture	[35,36,37,38,39,40]
Spanwise bending	Provides load-bearing deformation while maintaining wing integrity	Higher stiffness along principal longitudinal veins	[35,36]
Chordwise bending	Enables passive sectional deformation and camber	Lower directional stiffness and compliant cross-vein architecture	[35,36]
Wing folding	Enables compact storage and deployment	Folding lines, compliant joints, and mechanically programmed configurations	[31,38,41]

**Table 4:** Comparison of the most widely used experimental techniques for studying passive morphing, including the measured quantities, principal advantages, and current limitations.

Technique	Measures	Resolution	Advantages	Limitations
High-speed imaging	Wing kinematics	High temporal	Simple	2D information
Stereo imaging	3D motion	High	Full deformation	Complex calibration
Digital Image Correlation	Full-field displacement	High	Quantitative strain	Surface preparation
μCT	Internal geometry	Micrometer	Excellent structural detail	Static measurements
SEM	Surface morphology	Nanometer	High spatial resolution	Vacuum environment
CLSM	Material distribution	Micrometer	Resilin visualization	Small specimens

**Table 5:** Representative quantitative comparisons of computational approaches used to investigate insect-wing structural and aerodynamic behaviour. Values are reported together with the principal modelling conditions and should not be interpreted independently of those conditions.

Study	Approach	Wing/Conditions	Structural or Aerodynamic Assumptions	Representative Quantitative Result	Interpretation
Jin et al. [65]	FEM	Beetle wing; span ≈ 50 mm; chord ≈ 20 mm	Geometry divided into 48 regions to represent thickness variation	FEM bending response agreed with experimental bending test; exact percentage error not reported in accessible article summary	Demonstrates importance of geometrically resolved stiffness distribution
Darvizeh et al. [66]	FEM modal analysis	Dragonfly forewing; *f*_flap_ ≈ 27 Hz	Veins: 0.135 mm OD, 0.025 mm wall; membrane: 0.003 mm; *E* = 7.5 GPa, *ρ* = 1200 kg/m^−3^	Natural frequencies: 181.12, 313.28, 390.14, 446.31 Hz; *f*_flap_/*f*_1_ ≈ 0.15; magnification ≈ 1.02	Fundamental response dominated by bending; structural resonance far above normal flapping frequency
Fitzgerald et al. [67]	FSI: DNS vs. UVLM	Flexible flapping system	Viscous DNS compared with inviscid vortex-lattice formulation	UVLM reproduced many global trends but diverged where viscous effects were significant	Model fidelity required depends on whether global loads or detailed vortex physics are sought
Ishihara [68]	Strongly coupled FSI	Insect-inspired micro-wing; *f* = 428 Hz	Passive pitching generated by structural–fluid coupling	Tip/base displacement ratio ≈ 19; stroke ≈ 73°; passive pitch ≈ 62°	Resonance and FSI can generate insect-like motion without directly prescribing pitch
Ishihara & Onishi [69]	Monolithic FSI	Dynamically similar wing; *Re* = 819.8, *Ca* = 0.2420, *R_M_* = 0.001916	Flexible pixel-model wing	Tip feathering ≈ 31°; mean camber ≈ 5.4%; inertia contributed ≈ 11% of feathering and ≈15% of camber	Elastic–aerodynamic balance dominates passive deformation, with inertia enhancing stroke-reversal response
Ramamurti & Sandberg [75]	3D CFD	Dynamically scaled *Drosophila*; *Re* ≈ 136	Prescribed translational and rotational kinematics	Advanced rotation increased peak thrust; delayed rotation reduced it	Force production is strongly dependent on rotational timing
Ansari et al. [76]	Nonlinear vortex model	Hovering insect-like wing; *Re*∼200	2D unsteady wake model	Final-cycle lift: 0.3805 vs. 0.4081 N experimental (6.8%); thrust: 0.5904 vs. 0.6106 N (3.3%)	Reduced-order model captured calibrated force history with single-digit final-cycle error
Lankford et al. [72]	3D URANS CFD	MAV wing; *Re* = 25,000; 5 Hz; 107° stroke; pitch 40–60°	Rigid wing with experimental passive-pitch kinematics	50° case: max *L*/*D* ≈ 0.75, power loading 0.19 N/W; 40°: 0.16 N/W; 60°: 0.14 N/W	Aerodynamic performance and validation quality depend strongly on pitch kinematics

**Table 6:** Representative quantitative validation cases for analytical and computational models of insect-inspired flapping wings.

Study	Model Validated	Experimental Reference/Measurement	Conditions	Quantity Compared	Quantitative Agreement or Discrepancy
Darvizeh et al. [68]	FEM	Experimental dragonfly-wing vibration data	*f*_flap_ ≈ 27 Hz; isotropic *E* = 7.5 GPa model	Natural frequency/mode shape	Predicted *f*_1_ = 181.12 Hz; flapping frequency ≈ 15% of *f*_1_; reported agreement with experimental modal data
Ansari et al. [73]	Nonlinear unsteady aerodynamic model	Dickinson/Robofly force data	Hover; *Re*∼200	Mean lift and thrust	Full sequence: lift error 13%, thrust 7.2%; final cycle: lift 6.8%, thrust 3.3%
Ramamurti & Sandberg [72]	3D CFD	Dickinson et al. dynamically scaled *Drosophila* experiment	*Re* ≈ 136; mean chord 6.7 cm in scaled model	Thrust/drag histories and vortex development	Grid-independent solution; force trends reproduced; force peaks changed systematically with advanced/symmetric/delayed rotation
Lankford et al. [74]	3D URANS CFD	Force measurements + PIV	*Re* = 25,000; 5 Hz; 107° stroke; pitch 40°, 50°, 60°	Instantaneous forces, LEV evolution, power loading	Predicted forces generally within experimental uncertainty; strongest power-loading discrepancy at 40°; 50° CFD power loading 0.19 N/W
Xue et al. [77]	Coupled CFD–CSD/flexible multibody model	Free-flight measurements of DOVE FWMAV	Forward trimmed flight	Plunging acceleration and pitch oscillation	Study reports close agreement with slight residual discrepancies; numerical percentage error was not provided in the accessible published abstract

**Table 7:** Comparison of material systems considered for morphing-wing structures.

Material System	Principal Advantage	Main Limitations	Suitable Morphing Role	References
Variable-stiffness composites	Tailored anisotropy and bend–twist coupling	Manufacturing variability, delamination and fatigue	Passive load adaptation and stiffness tailoring	[117,118,121]
Multistable composites	Retain multiple shapes without continuous power	Sensitive switching loads and dynamic transitions	Shape locking and discrete passive morphing	[116,117]
Elastomeric skins	High strain capability and low deformation resistance	Crack sensitivity, aging and limited load-bearing capacity	Continuous flexible aerodynamic surfaces	[122]
Shape-memory alloys	High actuation energy density and compact integration	Hysteresis, thermal delay and energy consumption	Camber and twist actuation	[123]
Macro-fibre composites	Rapid distributed actuation and low mechanical complexity	Limited stroke, creep and out-of-plane sensitivity	Local camber control and vibration suppression	[123,124]
Shape-memory polymers and liquid-crystal elastomers	Programmable deformation and shape retention	Slow thermal response, limited load capacity and environmental sensitivity	Venation-inspired passive deformation	[120]
Heterogeneous metamaterials	Locally programmable stiffness and deformation	Complex design, manufacturing and validation	Venation-inspired passive deformation	[125,126]

**Table 8:** Comparative maturity of emerging research directions in bio-inspired morphing structures.

Emerging Direction	Principal Contribution to Morphing Systems	Current Maturity	Main Limitations and Research Priorities	References
AI-assisted modelling and design	Accelerates CFD/FSI prediction, structural optimization, shape selection, and control-policy development	Moderate for offline design and prediction; low-to-moderate for real-time autonomous morphing	Limited standardized datasets; poor extrapolation outside the training domain; uncertainty quantification; physical consistency; experimental and flight validation	[132,133,134]
Additive and 4D manufacturing	Enables distributed compliance, anisotropic stiffness, cellular reinforcement, integrated mechanisms, and spatial material grading	Moderate at component and wind-tunnel-model level	Manufacturing variability; surface quality; multi-material interfaces; fatigue; fibre-path control; repeatability and scalability	[132,133,134,135,136,137,138]
Digital twins and lifecycle modelling	Connects numerical models with real-time sensing for state estimation, adaptive control, damage detection, and model updating	Low-to-moderate; primarily numerical or subsystem-level	Real-time data assimilation; sensor durability; computational cost; model drift; incomplete damage and aging models; limited flight validation	[140,141]
Multifunctional materials and structures	Integrates load bearing, morphing, sensing, damping, energy harvesting, environmental protection, and damage tolerance	Moderate for individual functions; low for fully integrated multifunctional wings	Functional interference; added mass and complexity; durability; power management; repairability; environmental resistance	[142,143]
Planetary flight applications	Provides compact packaging, post-entry deployment, atmospheric adaptation, terrain interaction, and reduced actuator demand	Low; predominantly conceptual, numerical, or laboratory-scale	Low-density fluid–structure interaction; cryogenic and radiation resistance; dust; deployment reliability; autonomous operation; energy limitations	[144,145,146]

## Data Availability

All generated data are contained in the article or can be made available upon request.

## Abbreviations

The following abbreviations are used in this manuscript:

AI	artificial intelligence
BMP	bone morphogenetic protein
CAD	computer-aided design
CC BY	Creative Commons Attribution
CFD	computational fluid dynamics
CLSM	confocal laser scanning microscopy
CSD	computational structural dynamics
CT	computed tomography
Cv	Crossveinless
DEA	dielectric elastomer actuator
DIC	digital image correlation
DMD	dynamic mode decomposition
DPIV	digital particle image velocimetry
Dpp	Decapentaplegic
EAP	electroactive polymer
EGFR	epidermal growth factor receptor
FBG	fiber Bragg grating
FE	finite element
FEM	finite element method
FSI	fluid–structure interaction
Gbb	Glass bottom boat
Hh	Hedgehog
JNK	c-Jun N-terminal kinase
LEV	leading-edge vortex
MAV	micro air vehicle
MFC	Macro-Fiber Composite
PIV	particle image velocimetry
POD	proper orthogonal decomposition
PTA	phosphotungstic acid
PTFE	polytetrafluoroethylene
RANS	Reynolds-averaged Navier–Stokes
ROM	reduced-order model
SEM	scanning electron microscopy
SINDy	sparse identification of nonlinear dynamics
SMA	shape memory alloy
SMP	shape memory polymer
Sog	Short gastrulation
Tlr	Tolloid-related
UAV	unmanned aerial vehicle
μCT	micro-computed tomography
